# Tritrophic Interactions and Parasitoid Community Structure of Gall‐Inducing Herbivores in Arid Desert Ecosystems of Northern Xinjiang, China

**DOI:** 10.1002/ece3.74408

**Published:** 2026-10-08

**Authors:** Gaopeng Yu, Yuhao Nie, Caihong Gao, Hongying Hu

**Affiliations:** ^1^ Xinjiang Key Laboratory of Biological Resources and Genetic Engineering College of Life Science and Technology, Xinjiang University Urumqi Xinjiang China

**Keywords:** biological control, gall inducers, *Lycium ruthenicum*, *Scrobipalpa erichiodes*, tritrophic interactions

## Abstract

Tritrophic interactions among plants, gall‐inducing herbivores, and their parasitoids are critical components of arid desert ecosystems, yet their complex network structures, and evolutionary adaptations remain poorly understood. This study elucidates the ecological networks and life‐history strategies of three distinct gall inducers and their associated parasitoid communities on *Lycium ruthenicum*, a keystone shrub in Northern Xinjiang, China. First, we report the globally novel gall‐inducing behavior in the genus *Scrobipalpa*, identifying *Scrobipalpa erichiodes* (Lepidoptera: Gelechiidae) as the inducer of swollen twig galls, where gall morphology acts as a significant predictor of internal larval development. Alongside *S. erichiodes*, we characterized the phenological dynamics of *Aceria pallida* (leaf galls) and *Contarinia* sp. (flower bud galls). Furthermore, we recovered a highly complex parasitoid community comprising 13 species, establishing multiple new host records. The dominant parasitoids regulating these gall inducers were *Pholetesor teresitergum* , *Cirrospilus pictus*, and *Pseudotorymus jaapiellae*, respectively. Notably, the high dominance of *C. pictus* on the gall mite *A. pallida* represents an unusual cross‐guild host shift, highlighting the adaptive plasticity of natural enemies in resource‐heterogeneous desert environments. Finally, laboratory assays revealed a significant negative correlation between increasing temperatures (15°C–30°C) and the adult longevity of these dominant parasitoids. These findings not only provide novel insights into the evolutionary ecology of gall‐induction but also underscore the vulnerability of parasitoid‐mediated top‐down control to thermal stress. Ultimately, this research deciphers the complex tritrophic networks sustaining arid ecosystems, offering a theoretical foundation for predicting species interactions and community stability under changing climate conditions.

## Graphical abstract

This study systematically elucidates the complex tritrophic networks among the keystone shrub Lycium ruthenicum, three gall‐inducing pests, and 13 associated parasitoid species in the arid desert ecosystems of northern Xinjiang, China, reporting the globally novel stem gall‐inducing behavior of Scrobipalpa erichiodes. We characterize distinct phenological and population dynamics across the guilds and identify a specialized natural enemy community where Cirrospilus pictus exhibits an unusual, dominant cross‐guild host shift to regulate the leaf gall mite Aceria pallida. Furthermore, laboratory assays demonstrate a significant negative correlation between rising temperatures (15°C–30°C) and adult parasitoid longevity, highlighting the potential vulnerability of top‐down biological control to climate‐driven thermal stress in fragile environments.

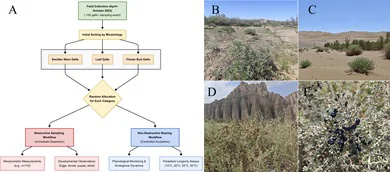

## 1 Introduction

*Lycium ruthenicum* Murray (Solanaceae: *Lycium*) is a perennial, salt‐tolerant shrub widely distributed in arid and semi‐arid regions (Peng et al. 2014). The genus *Lycium* comprises approximately 80 species globally, found across South America, North America, Australia, Eurasia, the Pacific Islands, and South Africa (Dhar et al. 2011). *L. ruthenicum* originated in the Americas and subsequently spread to Eurasia (Wang, Li, et al. 2018); in China, it is primarily distributed in the northern regions, with Xinjiang serving as one of the major cultivation centers (Rehman et al. 2024). Its fruits are rich in bioactive nutrients, including anthocyanins, polysaccharides, amino acids, and carotenoids, which exhibit antioxidant, anticancer, anti‐inflammatory, and hypoglycemic properties (Jeepipalli et al. 2026; Wang, Zeng, et al. 2018). It is also utilized in traditional Chinese medicine (Yao et al. 2018). As a keystone species in desert ecosystems, the plant possesses a robust root system that plays a critical role in windbreaks, sand fixation, and soil amelioration, acting as an essential barrier for maintaining ecological balance in arid zones. However, the recent rapid expansion of artificial cultivation in Xinjiang has led to monoculture patterns that disrupt the original ecological equilibrium, triggering frequent outbreaks of specialist gall inducers. Among these, gall inducers significantly constrain the sustainable development of the industry due to their concealed feeding habits, which cause leaf malformation, flower bud abortion, and even branch dieback.

Plant galls are abnormal vegetative growths or protrusions resulting from accelerated cell division and abnormal differentiation induced by the feeding or oviposition of organisms such as insects; these structures provide natural shelter for gall‐inducers, protecting them from parasitoids (Schultz et al. 2019; Stone and Schönrogge 2003). Currently reported gall‐inducing species on *L. ruthenicum* include the goji gall mite, *A. pallida* Keifer, and the goji gall midge, *Contarinia* sp. (Liu, Li, et al. 2016; Zhao et al. 2023). *Aceria pallida* infests leaves, flower buds, and tender shoots, causing deformities and flower abortion (Fu et al. 2021; Westphal et al. 1981). *Contarinia* sp. oviposits in flower buds, stimulating the formation of lantern‐shaped galls that prevent normal flowering and fruiting (Jiao et al. 2020). In addition, a highly concealed and novel galling phenomenon—characterized by swollen, club‐shaped galls on the tender stems of *L. ruthenicum*—has recently been observed in northern Xinjiang, though the specific ecological mechanisms and the identity of the inducer have remained unclear. In fragile arid desert ecosystems, where resources are severely constrained, tritrophic interactions among host plants, insect herbivores, and parasitoids are of profound ecological significance, acting as fundamental drivers of community stability and ecological resilience. Within these complex networks, top‐down suppression mediated by natural parasitoid communities serves as a vital biological mechanism regulating herbivore populations. Therefore, biological control through the application of parasitoids is a critical ecological strategy in agricultural and forestry gall inducer management. Parasitoids suppress target herbivore populations by ovipositing into or onto the host, where the hatching larvae consume the host to complete their development, offering both high efficiency and environmental friendliness (Zhou, Lu, et al. 2022; Zhou, Wu, et al. 2022). Compared to chemical control, biological control avoids residue pollution, delays the evolution of chemical pesticide resistance, maintains agroecological balance, and minimizes harm to non‐target organisms such as pollinators (Cusumano and Volkoff 2021). As natural enemies, promoting parasitoids not only improves control efficiency against major gall inducers but also supports green, sustainable prevention strategies, which is of profound significance for protecting biodiversity and maintaining ecosystem stability (Ye et al. 2024; Mansion et al. 2023). Despite the significant ecological and economic threats posed by these gall‐inducing herbivores on *L. ruthenicum*, critical knowledge gaps remain. Specifically, the composition of their associated parasitoid communities, the extent of host specificity or cross‐guild host shifts, and the spatiotemporal synchronization between gall inducers and their natural enemies are still poorly understood. Furthermore, while arid desert ecosystems are characterized by extreme midsummer heat, how such ambient thermal stress impacts adult parasitoid longevity and constrains their top‐down regulatory potential remains completely unexplored. To address these core ecological questions, this study systematically investigates the complex tritrophic networks involving *L. ruthenicum*, two previously known gall inducers ( *A. pallida* and *Contarinia* sp.), and the newly identified stem‐galler, *S. erichiodes* (Li and Bidzilya 2019) (Lepidoptera: Gelechiidae), whose novel gall‐inducing behavior is officially documented here for the first time. By investigating their associated parasitoid communities, this study aims to provide a theoretical foundation for the biological management of galling insects in arid desert ecosystems. Specifically, our primary objectives are to: (1) characterize the parasitoid community structure, dominance hierarchy, and host exclusivity; (2) identify potential cross‐guild host shifts among natural enemies adapting to resource‐heterogeneous environments; (3) evaluate the spatiotemporal emergence synchrony between gall inducers and their parasitoids; and (4) quantify the thermal responses of dominant parasitoids to assess the physiological vulnerability of top‐down biological control under changing climate conditions.

## 2 Methods

### 2.1 Study Sites

This study was conducted in 2023 in two representative distribution areas of *L. ruthenicum* in northern Xinjiang. The specific study sites were: (1) A desert plot near the 312 Township Road in Wujiaqu City (44°20′11.062″ N, 87°27′41.270″ E; Elevation: 450.9 m); (2) A plot in Manas County, Changji Prefecture (44°30′35.075″ N, 86°23′52.919″ E; Elevation: 398.5 m). Both sites represent typical arid desert habitats where *L. ruthenicum* is the dominant shrub species (Figure 1B–E). The plants were in good growth condition, and no chemical pesticides had been applied, ensuring the natural occurrence of gall inducer and parasitoid populations. Since both the Wujiaqu and Manas sites share highly similar climatic profiles, soil characteristics, and vegetation structures typical of northern Xinjiang desert ecosystems, and preliminary observations indicated no significant site‐specific differences in gall morphology or insect community composition, data from both sites were pooled for all subsequent analyses. This intentional pooling aims to increase statistical power and provide a more robust, generalized representation of the tritrophic interactions across the broader arid desert region, rather than focusing on localized micro‐site variations.

**FIGURE 1:**
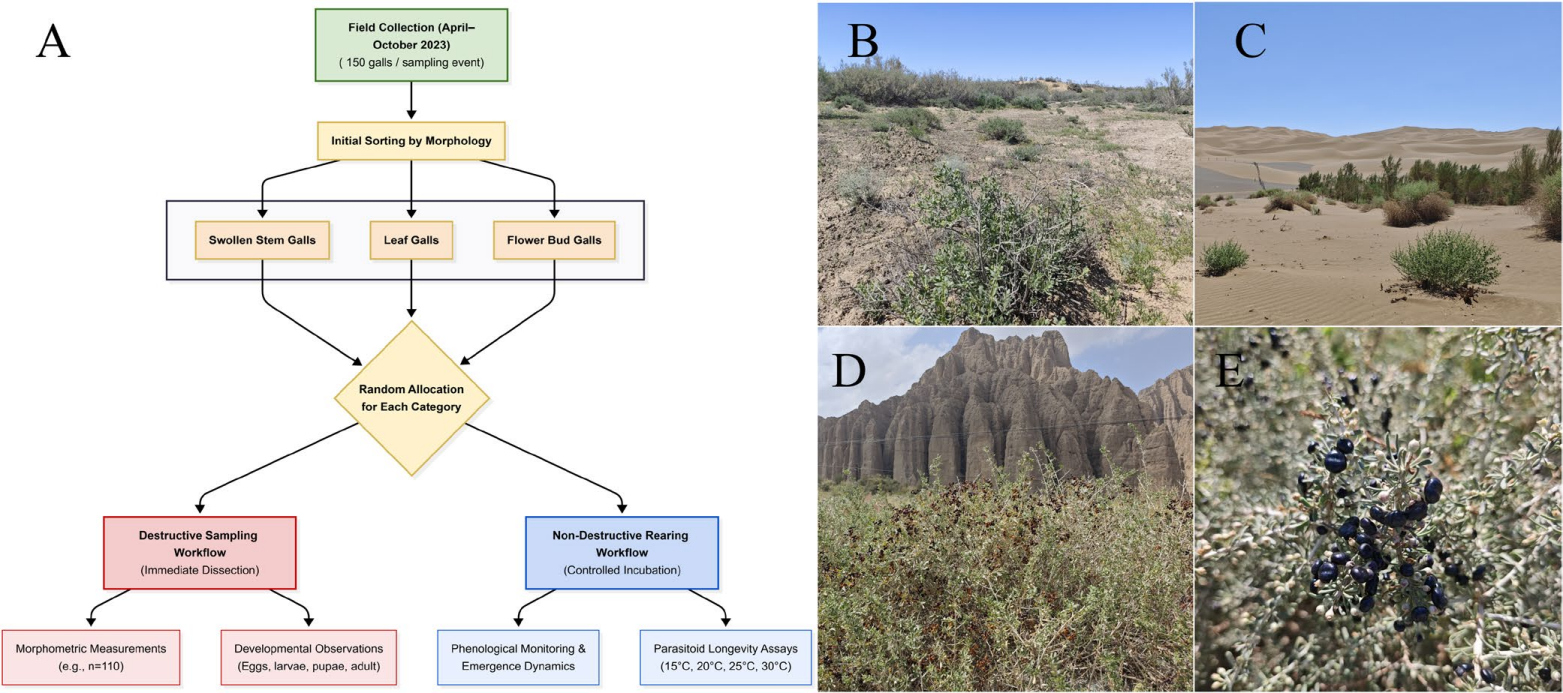
Experimental design, study site habitats, and host plant morphology of *L. ruthenicum*. (A) Schematic overview of the experimental design. (B, C) Representative field photographs showcasing the typical arid desert habitats of the study sites in northern Xinjiang. (D) Gross morphological appearance of the keystone host shrub, *L. ruthenicum*. (E) Detailed close‐up view of *L. ruthenicum* showing morphological features of leaves, shoots, and mature fruits.

### 2.2 Investigation of Biological Characteristics of Gall Inducers

Field sampling was carried out across the entire active growing season of *L. ruthenicum*, from April 20 to October 10, 2023. Sampling was conducted at approximately 10‐day intervals, resulting in a total of 18 sampling events. To ensure a balanced and highly reproducible sampling design, a 50 × 50 m plot was established at each of the two study sites, and sampling was conducted using a five‐point sampling method within 10 × 10 m subplots. During each sampling event, approximately 150 galls were randomly collected from each site, covering sequential host phenological stages: spring budding and primary shoot elongation (April–May), flowering and fruit development (May–August), and late‐season maturation (September). Because the three target gall‐inducing species exhibit distinct seasonal phenologies and generation times, this uninterrupted 10‐day interval sampling design was specifically implemented to span their entire active periods without temporal bias. This standardized temporal coverage ensured not only the comprehensive collection of galls across all major developmental phases—from initial induction to final maturation—but also the accurate capture of discrete adult emergence peaks, generational overlaps, and specific parasitic windows for the entire tritrophic community.

Upon transfer to the laboratory, all collected galls were initially sorted into three distinct categories based on their conspicuous macroscopic morphologies: swollen stem galls, leaf galls, and flower bud galls. To systematically assign these samples, the sorted galls within each category were randomly divided into two parallel workflows: a destructive sampling workflow and a non‐destructive rearing workflow (Figure 1A).

For the destructive workflow, a designated subset of fresh galls (including the 110 swollen galls used for morphometrics) was immediately dissected. The morphological characteristics and developmental stages (eggs, larvae, pupae, and adults) of *S. erichiodes*, *A. pallida* , and *Contarinia* sp. were observed and recorded using a Nikon Eclipse optical microscope. Additionally, their internal gall dimensions and life histories were documented using a Nikon SMZ25 stereomicroscope coupled with a DS‐U3 image synthesis system.

For the non‐destructive rearing workflow, the remaining intact galls were assigned to glass test tubes (1.5 cm × 10 cm). For each rearing session, 15 groups were established, with 10 galls per group. Rearing conditions were strictly maintained across four temperature gradients (15°C, 20°C, 25°C, and 30°C) with a relative humidity of 70% and a photoperiod of 14:10 (L:D). These intact samples were used exclusively for continuous phenological monitoring, assessing adult emergence dynamics, and collecting newly emerged parasitoids for temperature‐dependent longevity experiments. This strict bifurcation ensured that samples utilized for internal morphological observations did not interfere with the adult emergence and survival assays.

### 2.3 Morphometric Measurements of Swollen Galls

Galls collected on different sites were dissected using pruning shears. The swollen, club‐shaped galls were cut along their longitudinal axis and examined under a Nikon SMZ25 stereomicroscope. The following morphometric parameters were measured: gall chamber length (GL), gall chamber width (GW), gall thickness (GT), larval body length (LL), and larval body width (LW). A total of 110 samples were measured. These 110 gall samples were randomly and evenly selected from collections made during the peak gall‐formation period (mid‐to‐late May), with an equal number of samples (55 galls) derived from each of the two study sites to prevent site‐specific bias.

The gall chamber was approximated as an ellipsoid, and its volume was calculated using the ellipsoid volume formula: Gall Chamber Volume = (4 × GL × GW^2^)/3 (Figure 2).

**FIGURE 2:**
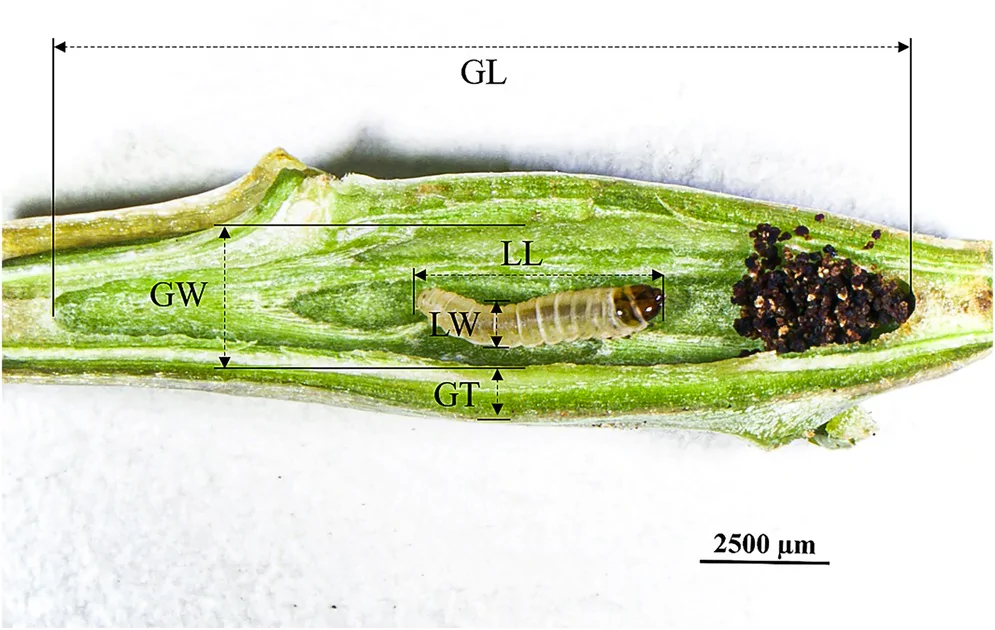
Longitudinal section of a swollen stem gall induced by *Scrobipalpa erichiodes* on *Lycium ruthenicum*.

### 2.4 Investigation of Parasitoid Biological Characteristics

Newly emerged parasitoids from the galls were transferred to fresh glass test tubes. A cotton strip soaked in a 15% honey solution was placed at the opening of each tube to provide supplementary nutrition, following established laboratory rearing protocols for evaluating parasitoid longevity (Fadamiro and Heimpel 2001; Siekmann et al. 2001), and the tube was then sealed with gauze. Rearing was conducted under conditions of 65% RH and a 14:10 (L:D) photoperiod across four temperature gradients: 15°C, 20°C, 25°C, and 30°C. For each dominant parasitoid species, approximately 10 newly emerged adults were tested per temperature treatment. Parasitoid longevity was monitored every 24 h, with the honey solution replenished as needed. Mortality was strictly defined as a complete loss of mobility and no response to gentle mechanical stimulation with a fine camel‐hair brush, at which point the survival time (in days) was formally recorded.

A subset of galls was dissected to observe and photograph the parasitism status of parasitoids within the galls. Following standard ecological methods for relative abundance evaluation (Magurran and Henderson 2003), community dominance and functional group dominance were calculated for each gall type to elucidate the parasitoid community structure, using classification criteria established in recent parasitoid community studies (Zhao et al. 2021). In this study, parasitoid functional groups (guilds) were delimited based on their specific host associations. Specifically, three distinct functional groups were defined corresponding to the three gall‐inducing herbivores: (1) the *S. erichiodes*‐associated parasitoid guild, (2) the *A. pallida* ‐associated parasitoid guild, and (3) the *Contarinia* sp.‐associated parasitoid guild. The calculations were performed as follows: Community dominance = (Number of individuals of a specific species/Total number of individuals of all species in the gall community) × 100%. Functional group dominance = (Number of individuals of a specific species/Total number of individuals within its respective functional group) × 100%.

### 2.5 Species Identification and Voucher Deposition

The identification of gall inducers and their associated parasitoids was conducted based on detailed morphological examinations. Adult and immature specimens were examined under a Nikon SMZ25 stereomicroscope. The gall‐inducing herbivores were identified using traditional morphological characteristics and authoritative species diagnoses: *S. erichiodes* was identified based on external morphological features of adults and developmental stages (Bidzilya and Li 2010); the flower bud gall midge collected in this study represents a single morphospecies within the genus *Contarinia* (designated hereafter as *Contarinia* sp.). *Contarinia* sp. was determined following the diagnostic characteristics described by Jiao et al. (2020); and *A. pallida* was verified using standard acarological keys (Kuang et al. 2005). Parasitoid wasps were identified to the species level using corresponding regional taxonomic keys for Chalcidoidea and Ichneumonoidea (Zhu et al. 2002; Liu, He, and Chen 2016). When necessary, challenging taxa were further verified through consultation with relevant taxonomic experts. Voucher specimens of all identified gall inducers and parasitoids, along with galled plant materials, were properly prepared, labeled, and deposited in the Insect Collection of the College of Life Science and Technology, Xinjiang University, Urumqi, China.

### 2.6 Data Analysis

All data were initially compiled using Microsoft Excel 2019. Statistical analyses were performed using IBM SPSS Statistics 20.0. First, data normality and homoscedasticity were assessed using the Shapiro–Wilk test and Levene's test, respectively. For continuous morphometric and developmental data following a normal distribution, one‐way Analysis of Variance (ANOVA) followed by Tukey's HSD post hoc tests were utilized to compare differences among temperature treatments. Pearson correlation analysis was conducted to investigate the linear relationship between gall morphology and larval size, with the significance level set at *α* = 0.05.

Crucially, because adult parasitoid longevity represents time‐to‐event data, survival analyses were employed to provide a more robust analytical framework for evaluating thermal stress. Kaplan–Meier survival estimators were used to generate survival curves for each species across the four temperature gradients (15°C, 20°C, 25°C, and 30°C). Subsequently, Log‐rank (Mantel‐Cox) tests were conducted to statistically compare the survival distributions among the different temperature treatments. Correlation heatmaps were generated using the Chiplot online platform (https://www.chiplot.online), and all other figures were plotted using Origin 2021.

## 3 Results

### 3.1 Gall Induction Characteristics and Biological Traits

#### 3.1.1 Correlation Analysis

The correlation between the dimensions of the swollen, club‐shaped galls and larval morphometrics was analyzed. Normality tests confirmed that the data for all five measured traits followed a normal distribution. No significant correlation was observed between gall chamber length and width, whereas a significant correlation was found between gall chamber length and larval body length (Table 1). All other measured indices exhibited highly significant linear correlations, suggesting that external gall morphology can be used to infer the developmental status of the larvae within (Figure 3).

**TABLE 1:** Statistical significance (*p*‐values) of Pearson correlation analyses among swollen gall morphometrics and *Scrobipalpa erichiodes* larval dimensions.

	CL	CW	CV	GT	LL	LW
CL	—	0.158	0.000	0.002	0.028	0.000
CW	0.158	—	0.000	0.000	0.000	0.000
CV	0.000	0.000	—	0.007	0.001	0.004
GT	0.002	0.000	0.007	—	0.000	0.000
LBL	0.028	0.000	0.001	0.000	—	0.000
LBW	0.000	0.000	0.004	0.000	0.000	—

*Note:* |*r*| < 0.3 weakly correlated; 0.5 > |*r*| ≥ 0.3 poorly correlated; 0.8 > |*r*| ≥ 0.5 moderately correlated; |*r*| ≥ 0.8 strongly correlated.

Abbreviations: CL, gall chamber length; CV, gall chamber volume; CW, gall chamber width; GT, gall thickness; LL, larval body length; LW, larval body width.

**FIGURE 3:**
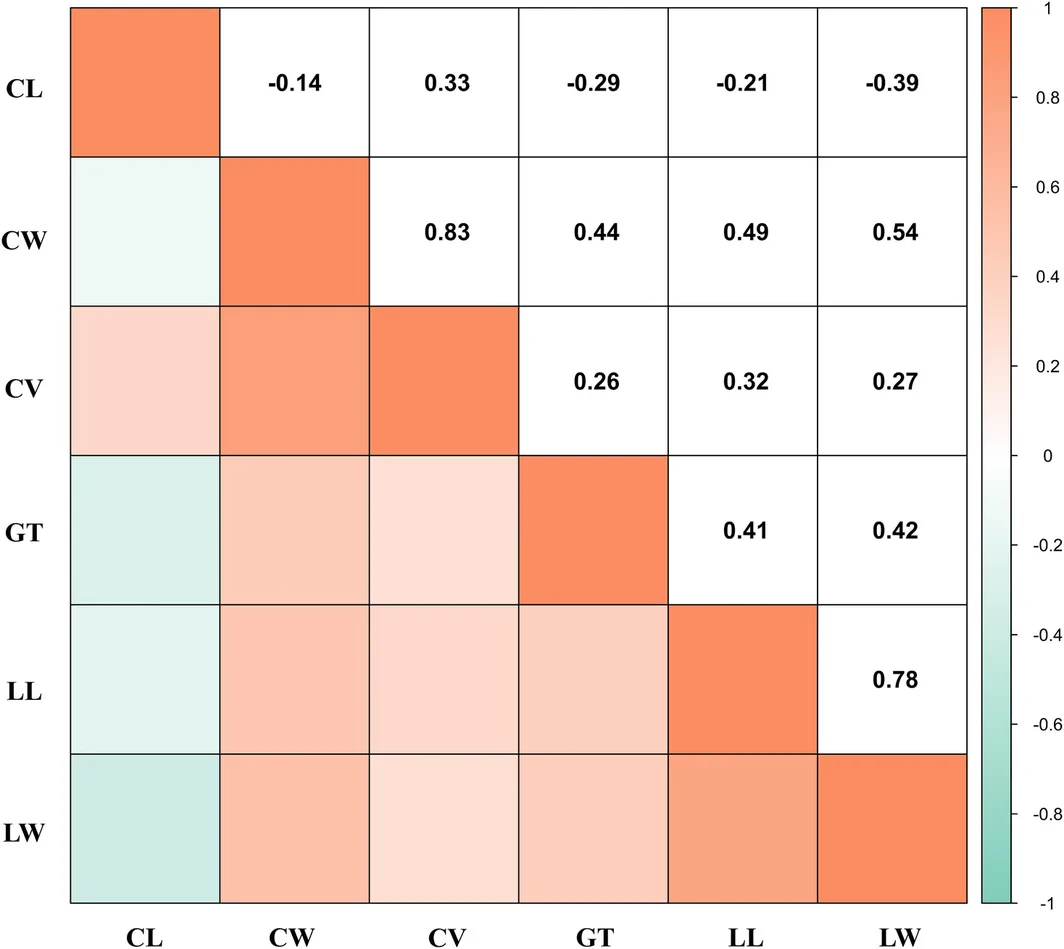
Correlation matrix heatmap between swollen gall morphometrics and *S. erichiodes* larval dimensions on *L. ruthenicum*. Numerical values inside the matrix represent Pearson correlation coefficients (*r*), with background colors indicating correlation direction and strength. Statistical significance levels are indicated by asterisks: *p < 0.05, **p < 0.01, ***p < 0.001. Correlation thresholds: (|*r*| < 0.3, weak correlation; 0.3 ≤ |*r*| < 0.5, low correlation; 0.5 ≤ |*r*| < 0.8, moderate correlation; |*r*| ≥ 0.8, strong correlation). CL, gall chamber length; CV, gall chamber volume; CW, gall chamber width; GT, gall thickness; LL, larval body length; LW, larval body width.

Weak negative correlations were observed between gall chamber length and gall thickness, larval length, and larval width (Figure 3). Conversely, weak positive correlations were found between chamber volume and both gall thickness and larval width. Similarly, weak positive correlations were identified for the following pairs: chamber width versus gall thickness and larval length; chamber volume versus larval length; and gall thickness versus larval length and width. Notably, moderate positive correlations were observed between chamber width and larval width, as well as between larval length and larval width.

#### 3.1.2 Gall Induction Characteristics and Biological Traits of *Scrobipalpa erichiodes*

Our study reveals that overwintering adults of *Scrobipalpa erichiodes* oviposit under the bark of tender *L. ruthenicum* shoots. Upon hatching, the larvae bore into the stems, stimulating tissue hypertrophy that results in hollow, club‐shaped galls. Histological observations indicate that while the epidermal cells of the galls show no significant morphological differences compared to normal branches, the pith cells undergo continuous differentiation into abundant, small, and densely packed nutritive cells consumed by the larvae. This process eventually forms a single chamber in the center of the stem (Figure 4).

**FIGURE 4:**
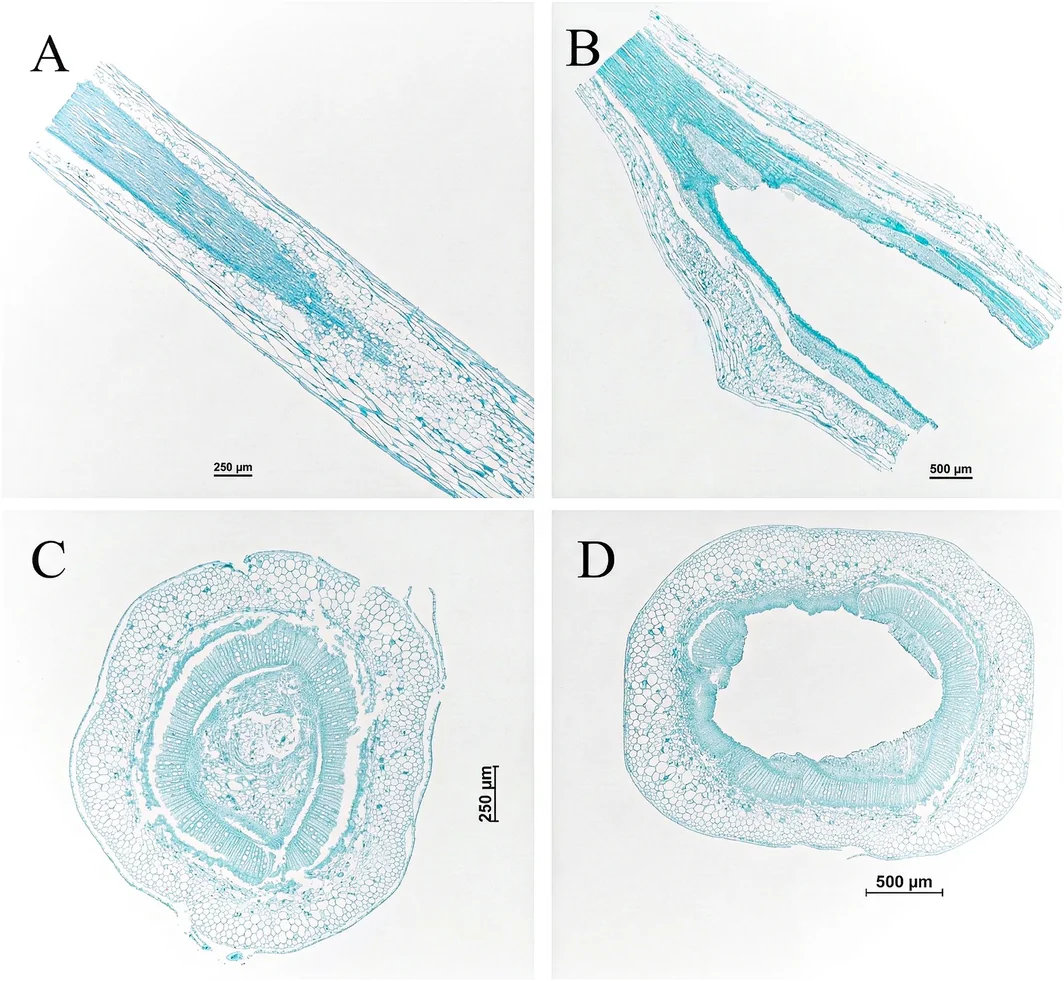
Paraffin anatomical sections of *L. ruthenicum* swollen galls. (A) Longitudinal section of normal branch; (B) Longitudinal section of swollen gall; (C) Transverse section of normal branch; (D) Transverse section of swollen gall.

Morphological observations of the developmental stages indicate that the eggs (Figure 5A) are spindle‐shaped, milky white, approximately 1 mm in length, with yellowish tips. The larvae (Figure 5B,C) pass through four instars; neonates are pale yellowish‐green with a pale brown dorsal stripe, whereas mature larvae measure 7–10 mm, turn pale yellow, and exhibit pale red dorsal striations on the trunk. The pupae, including the pre‐pupa and post‐pupa stages (Figure 5D,E), are 4–6 mm long, conical, initially pale yellow turning brown, and are enclosed in white cocoons, typically attached to withered leaves. The adults (Figure 5F,G) appear pale grayish‐brown, with a body length of 5–8 mm and a wingspan of 12–16 mm. The forewings (Figure 5H) are yellowish‐brown with dark brown spots, while the hindwings are pale yellowish‐brown with long cilia. The head in lateral view (Figure 5I) reveals filiform antennae consisting of 37–45 flagellomeres, with the labial palpi curving upward beyond the vertex. The abdomen is fusiform and marked with inverted “V”‐shaped patterns.

**FIGURE 5:**
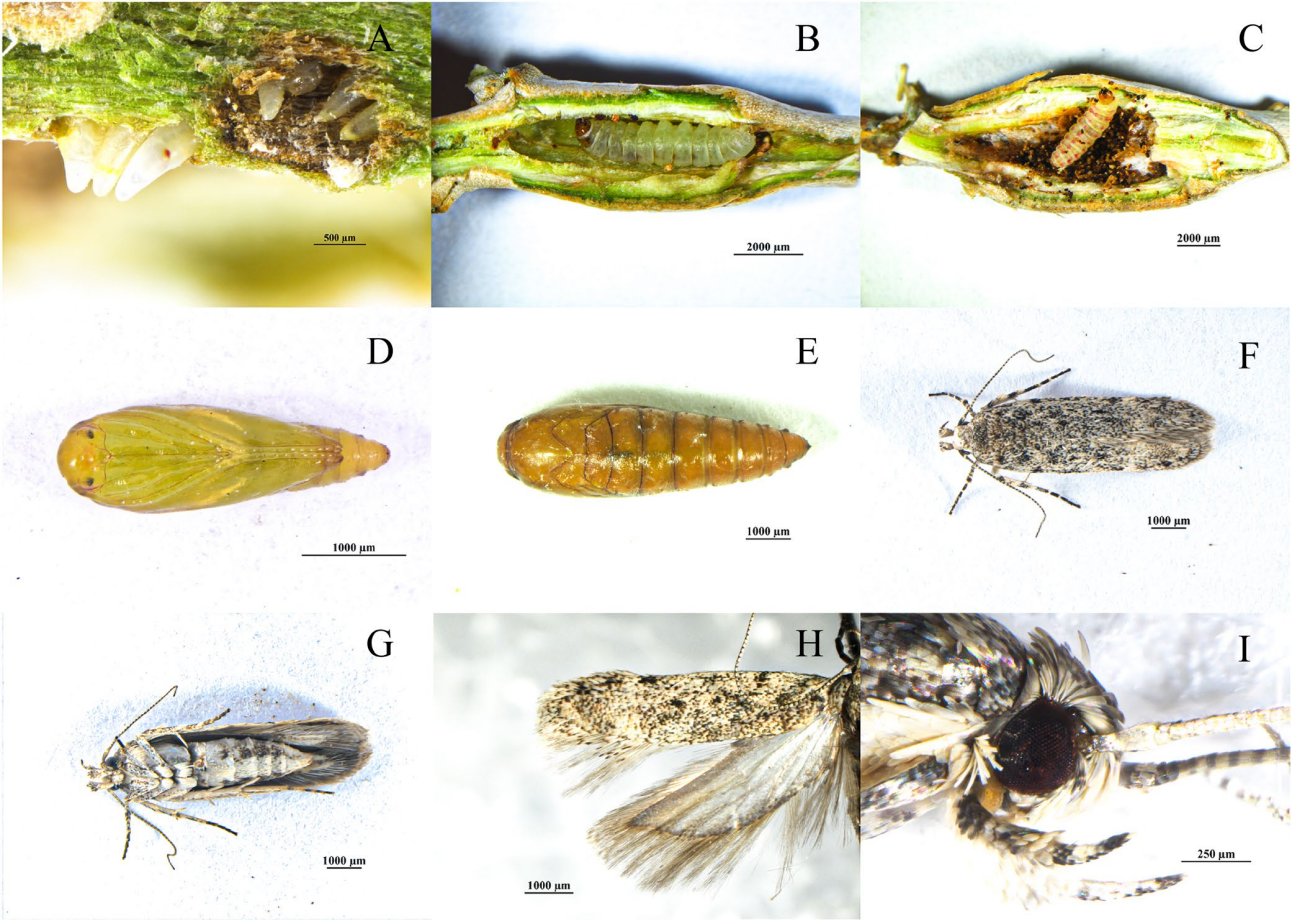
Morphological characteristics of *Scrobipalpa erichiodes* across developmental stages. (A) Egg; (B, C) Larvae; (D) Pre‐pupa; (E) Post‐pupa; (F) Adult (dorsal view); (G) Adult (ventral view); (H) Forewing; (I) Head (lateral view).

In the Wujiaqu region of Xinjiang, *S. erichiodes* completes three generations per year (Table 2). Overwintering adults emerge in late April, and first‐generation larvae begin infesting tender shoots in mid‐May, with peak gall formation occurring in mid‐to‐late May. Three adult emergence peaks occur annually: the first in early June, corresponding to the peak period of fruit‐boring damage, and the second from late July to early August, followed by a second peak of fruit infestation in late August. The emergence of third‐generation adults is minimal. Notably, a high parasitism rate was observed in larvae during August and September, and some individuals failed to emerge after pupation, leading to a population decline. From mid‐to‐late September onwards, the gall inducer overwinters as pupae within cocoons in branch crevices.

**TABLE 2:** Life history table of *S. erichiodes*.

	April		May			June			July			August			September			November–March
	M	L	E	M	L	E	M	L	E	M	L	E	M	L	E	M	L	
OG	※	※	※															
G1			○	○	○	○	○											
G2							●	●	●	●	●	●						
G3											◇	◇	◇	◇	◇	◇		
OG																	※	※※※※

*Note:* ※OG: Overwintering generation; ○G1: First generation; ●G2: Second generation; ◇G3: Third generation.

Abbreviations: E, Early; L, Late; M, mid.

#### 3.1.3 Gall Induction Characteristics and Biological Traits of *Aceria pallida*

*Aceria pallida* preferentially infests differentiating tender leaves of *L. ruthenicum*. At the infestation site, epidermal cells form a small protrusion, serving as an entry channel for the mites. Following invasion, the palisade and spongy mesophyll tissues surrounding the mite degenerate (Figure 6), creating a primordial gall chamber. Initially, the mite population within the chamber is low. A layer of nutritive tissue, composed of small, densely packed parenchyma cells, differentiates around the chamber to sustain the mites and associated inquilines. As the gall develops, although the overall chamber volume changes minimally, the mite population increases sharply. Concurrently, the nutritive tissue thickens and projects inward, partitioning the chamber into multiple interconnected compartments, thereby significantly increasing the internal surface area.

**FIGURE 6:**
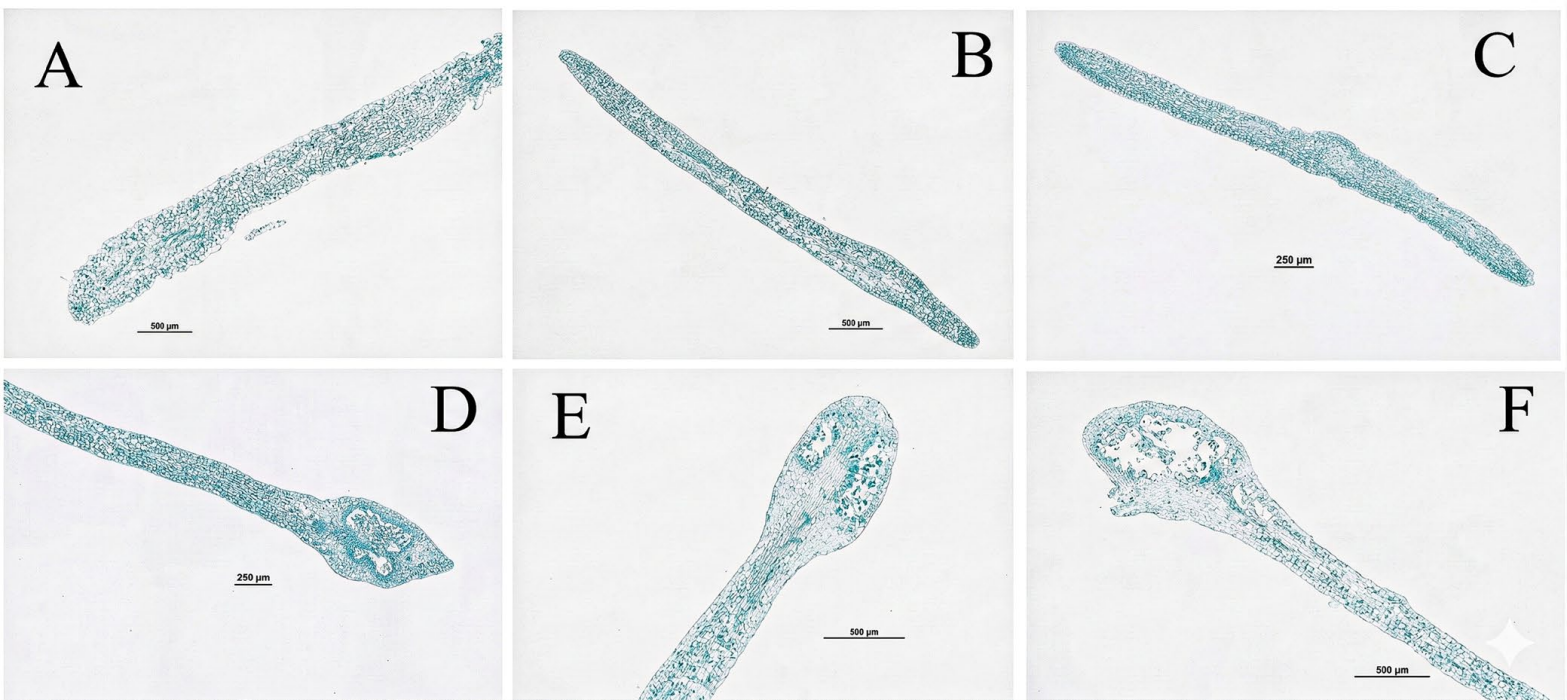
Paraffin anatomical sections of *Lycium ruthenicum* leaf galls induced by *Aceria pallida*. (A) Normal leaf; (B, C) Early leaf gall; (D) Middle leaf gall; (E, F) Late leaf gall.

Morphological observations of the developmental stages on *L. ruthenicum* indicate that the eggs (Figure 7A) are translucent white and subspherical, with a diameter of approximately 30 μm. The nymphs (Figure 7B) range from white to pale yellow, are semi‐translucent and fusiform, and resemble adults in body length and morphological differentiation. The adults (Figure 7C) measure 0.1–0.3 mm in length, vary from pale yellow to orange, and exhibit a long, conical, “carrot‐like” shape that curves slightly downward. The gnathosoma is broad and short, projecting forward like a beak. Adults possess two pairs of legs, and the opisthosoma bears 53–54 annuli.

**FIGURE 7:**
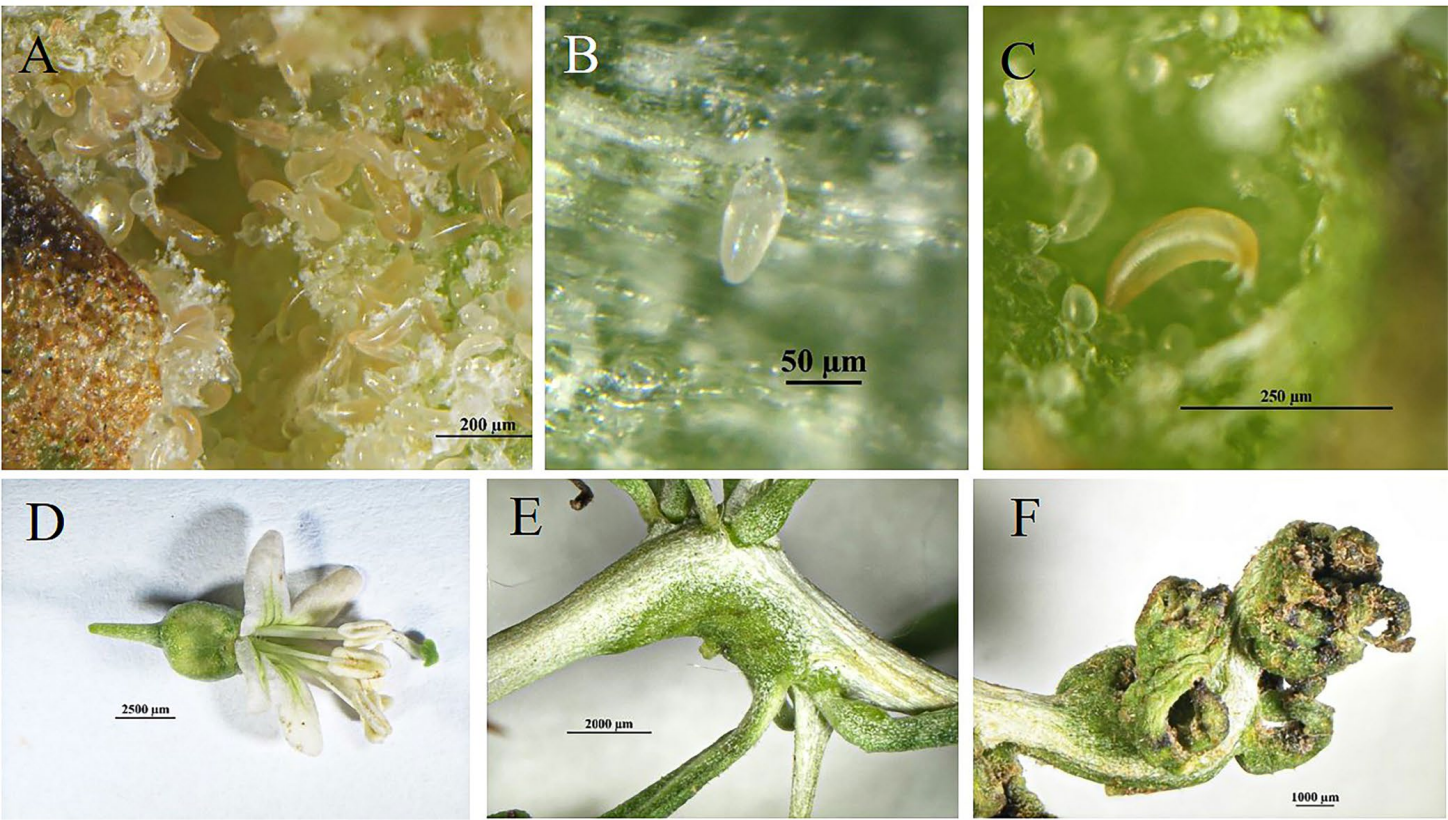
Developmental stages and damage symptoms of *Aceria pallida*. (A) Anatomy of leaf gall showing eggs; (B) Nymph; (C) Adult; (D) Damage to flower buds; (E, F) Damage to vegetative shoots.

Phenologically, *Aceria pallida* is multivoltine on *L. ruthenicum*, producing over ten generations annually with severe overlapping (Table 3). In northern Xinjiang, overwintering mites resume activity in late April as the host plant buds and flushes, infesting new leaves and tender tissues. Infestation occurs throughout the growing season, peaking in severity during June and July. Damage rates decline in September, and by late October, the mites enter dormancy, ceasing damage. The overwintering generation primarily shelters in branch crevices or axillary buds of infested shoots.

**TABLE 3:** The annual life history of *A. pallida* .

	April		May			June			July			August			September			October			November–March	
	M	L	E	M	L	E	M	L	E	M	L	E	M	L	E	M	L	E	M	L		
OG	※	※																				
Egg		○	○	○	○	○	○	○	○	○	○	○	○	○	○	○	○	○				
YM				●	●	●	●	●	●	●	●	●	●	●	●	●	●	●	●			
AM				◇	◇	◇	◇	◇	◇	◇	◇	◇	◇	◇	◇	◇	◇	◇	◇	◇		
OG																				※	※	※

*Note:* ※OG: Overwintering generation; ○: eggs; ●YM: young mites; ◇AM: adult mites.

Abbreviations: E, Early; L, Late; M, mid.

The mites primarily feed on leaf sap, stimulating tissue hyperplasia to form galls. Initial symptoms appear as yellow‐green spots on leaves, which subsequently develop into mole‐like protrusions that darken over time. As the mites feed and reproduce, these protrusions expand into hollow, enclosed galls (typically ~2 mm in diameter) where the mites breed through successive generations. Upon maturity, the gall epidermis ruptures, allowing adults to escape and disperse. Heavy infestation leads to leaf chlorosis, desiccation, and premature abscission, severely impairing the plant's photosynthetic capacity. In addition to leaves, the mites attack tender buds and calyces (Figure 7D) as well as tender shoots (Figure 7E,F), causing shoot distortion, calyx hypertrophy, and flower bud abortion. In severe cases, high gall density on a single leaf results in the fusion of individual galls into large, irregular masses.

#### 3.1.4 Gall Induction Characteristics and Biological Traits of *Contarinia* sp.

Adults of *Contarinia* sp. oviposit within the flower buds. Upon hatching, the larvae feed on the internal tissues, stimulating hyperplasia and hypertrophy of the cells at the base of the corolla (Figure 8), which expands to form a gall. While the morphology of the calyx remains largely unchanged, the base of the corolla turns purple, the middle‐to‐upper sections appear green, and the tip occasionally exhibits a yellowish hue. The galls are typically inhabited by multiple larvae, averaging approximately 10 individuals per gall. Both the larval and pupal stages are completed within the enclosed flower bud gall.

**FIGURE 8:**
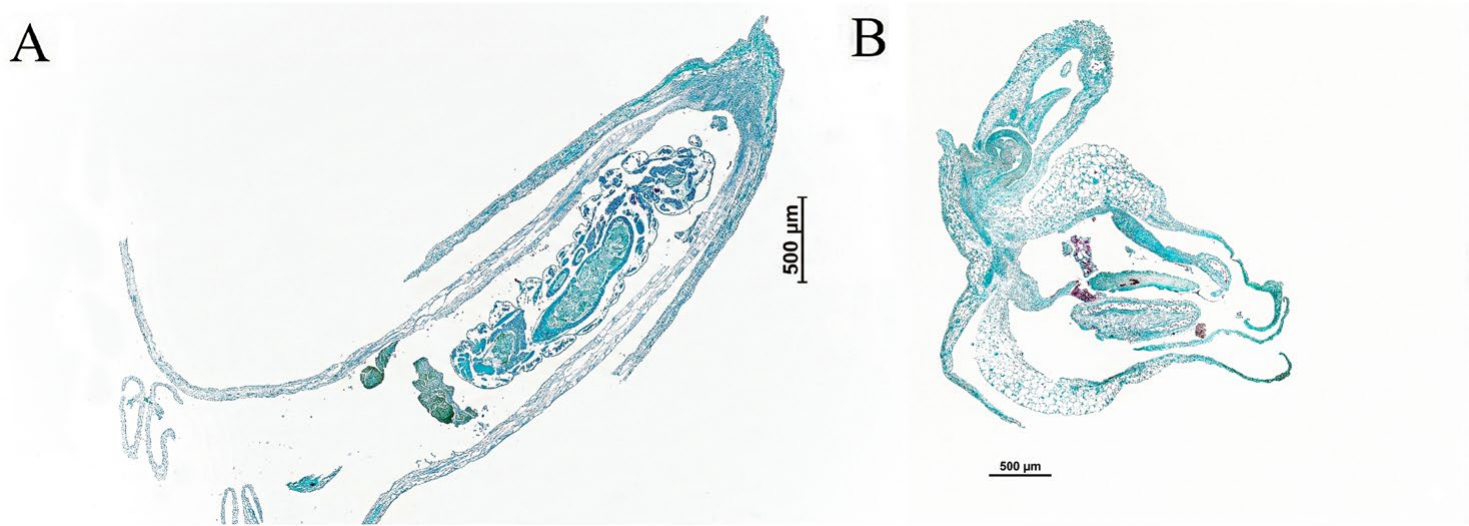
Paraffin anatomical sections of *Lycium ruthenicum* flower buds. (A) Normal flower bud; (B) Flower bud gall induced by *Contarinia* sp.

Morphological observations of the developmental stages indicate that the eggs are white, nearly translucent, and elliptical. The larvae (Figure 9A) measure approximately 2 mm in length. Neonates are yellowish‐white with a visible yellow alimentary canal and internal white adipose granules; as they develop into the pre‐pupa stage (Figure 9B), they transform into pale yellow, maggot‐like forms with increased adipose tissue and distinct segmentation. The pupae (Figure 9C,D) are approximately 1.5 mm long, initially yellow‐brown and darkening over time; they possess two distinct brown cephalic processes on the vertex, with visible antennae, wing pads, and legs appressed to the ventral surface. The adults (Figure 9E,F) have a body length of about 3 mm, appearing initially white upon emergence and gradually darkening to pale yellow.

**FIGURE 9:**
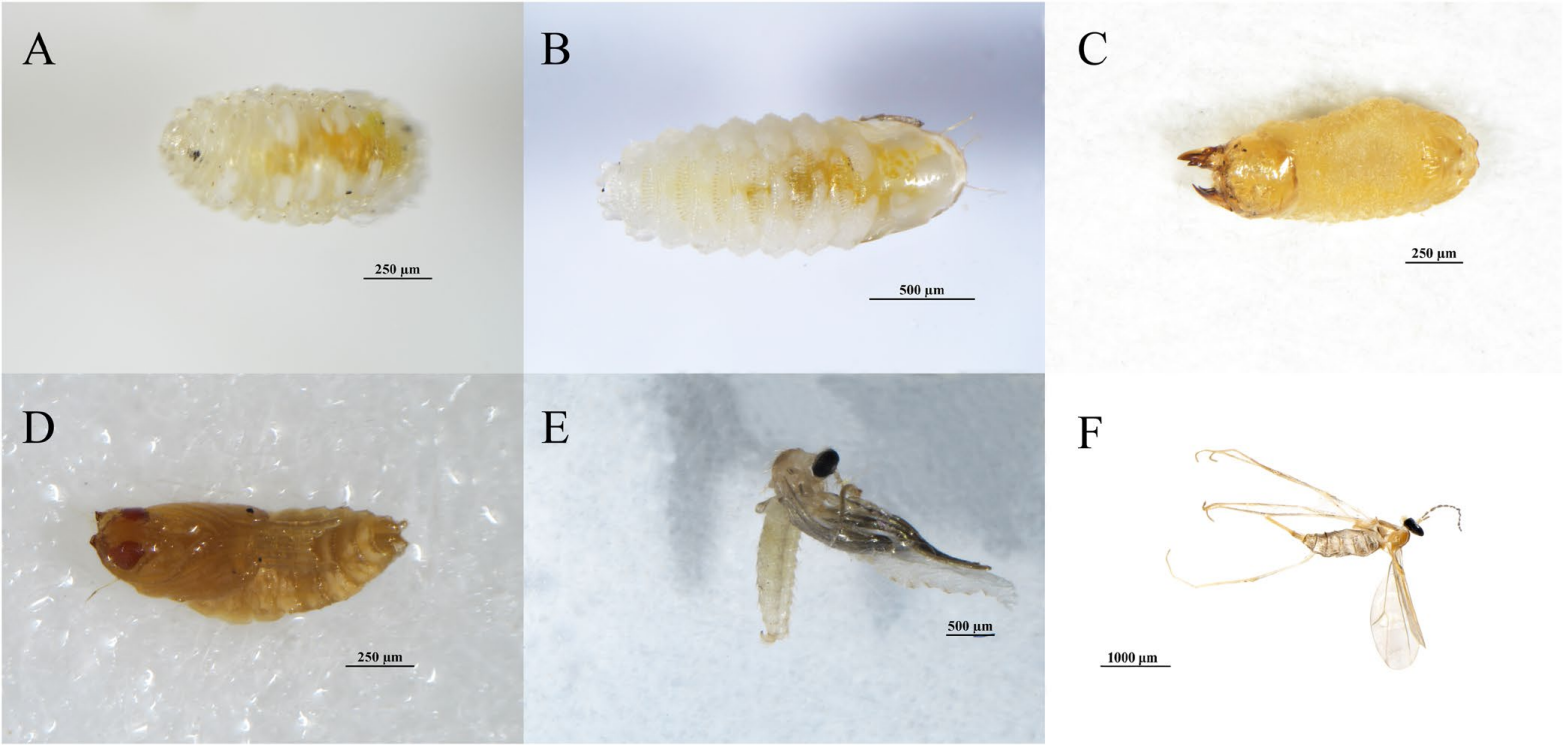
Developmental stages of *Contarinia* sp. (A) Larva; (B) Pre‐pupa; (C, D) Pupal stage; (E) Emergence; (F) Adult.

Phenologically, *Contarinia* sp. completes five generations annually in the Changji region (Table 4), with the first and fourth larval generations causing the most severe damage. Overwintering mature larvae pupate in mid‐April of the following year, with adults emerging in early May to oviposit and infest flower buds. Second‐generation larvae pupate in early June, with adults emerging in mid‐to‐late June. Third‐generation adults emerge in mid‐July. The fourth larval generation inflicts significant damage in mid‐to‐late August, while the fifth generation causes relatively minor damage. The gall inducer overwinters as mature larvae in the soil surrounding the host plants.

**TABLE 4:** The annual life history of *Contarinia* sp.

	April		May			June			July			August			September			October–March
	M	L	E	M	L	E	M	L	E	M	L	E	M	L	E	M	L	
OG	※	※	※															
G1			○	○	○	○												
G2						●	●	●	●									
G3									◇	◇	◇							
G4											▽	▽	▽	▽	▽			
G5														☆	☆	☆	☆	
OG																	※	※※※

*Note:* ※OG: Overwintering generation; ○G1: First generation; ●G2: Second generation; ◇G3: Third generation; ▽G4: Fourth generation; ☆G5: Fifth generation.

Abbreviations: E, Early; L, Late; M, mid.

The infestation period spans from early May to mid‐September. Females oviposit inside the flower buds, where larval feeding induces malformation and swelling of the corolla, resulting in characteristic “lantern‐shaped” galls. Once galled, the flower buds fail to develop and set fruit, directly correlating the infestation rate with yield loss. Field observations indicate that damage peaks occur primarily in late May and mid‐to‐late August, corresponding to the periods of highest gall density.

#### 3.1.5 Population Dynamics and Life Span Analysis

To elucidate the seasonal emergence rhythms of the three gall inducers, this study systematically investigated and compared the adult emergence dynamics of *Contarinia* sp., *A. pallida* , and *S. erichiodes* (Figure 10). The results indicated that *Contarinia* sp. exhibited a primary emergence peak in June, representing the period of highest population intensity and maximal adult output. This was followed by a decline and a subsequent, weaker peak in August, suggesting the occurrence of a distinct partial generation in late summer. Similarly, *A. pallida* reached its first major peak in early June, followed by a second high‐intensity peak from late July to early August, indicating significant population replenishment and a second wave of adult emergence within the same year. In contrast, *S. erichiodes* displayed a distinct trimodal emergence pattern that closely corresponds to its three‐generation annual life cycle, although the population intensity varied sharply among successive generations. The first emergence peak, occurring in early June, was the highest and most pronounced, representing the primary wave of adult output. The second peak, spanning from late July to early August, was substantially lower in amplitude. The third peak was extremely weak and nearly negligible, reflecting the minimal emergence of third‐generation adults. Overall, the progressive decline across these three emergence peaks directly reflects the changing scale of adult output and population intensity throughout the season, clearly differentiating the generation frequency and seasonal replenishment capabilities among the three taxa.

**FIGURE 10:**
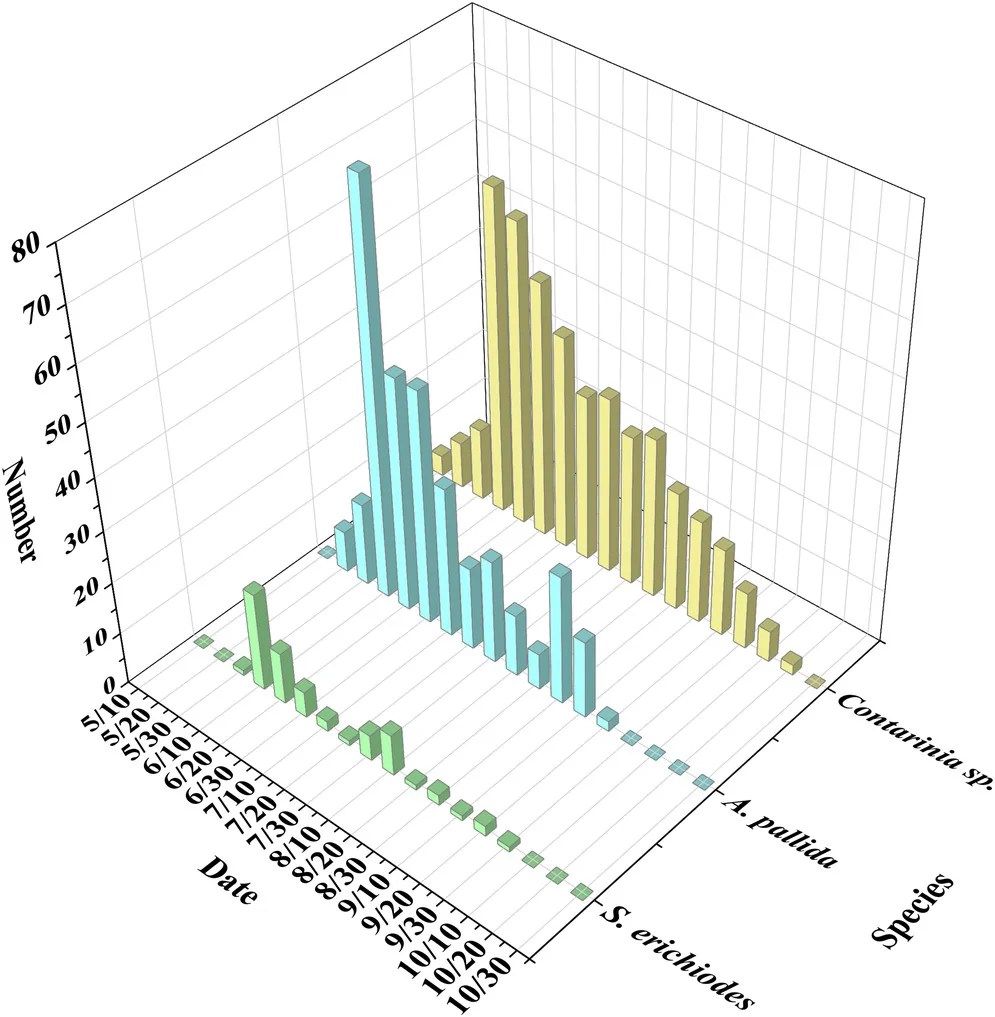
Seasonal adult emergence dynamics of the three gall inducers on *Lycium ruthenicum*.

Regarding the effect of temperature on adult longevity, species‐specific variations were observed within the 15°C–30°C range (Figure 11). For *S. erichiodes*, adult longevity was maximized at 25°C, whereas both *A. pallida* and *Contarinia* sp. exhibited their longest lifespans under cooler conditions (15°C). Despite these different thermal optima, extreme high temperature (30°C) consistently and significantly compressed the lifespan of all three species. Kaplan–Meier survival analysis demonstrated that thermal gradients significantly altered the survival probability distributions of *S. erichiodes* (Log‐rank test: *χ* ^2^ = 154.12, *p* < 0.001), *A. pallida* (Log‐rank test: *χ* ^2^ = 180.22, *p* < 0.001), and *Contarinia* sp. (Log‐rank test: *χ* ^2^ = 140.87, *p* < 0.001) (Figure S1).

**FIGURE 11:**
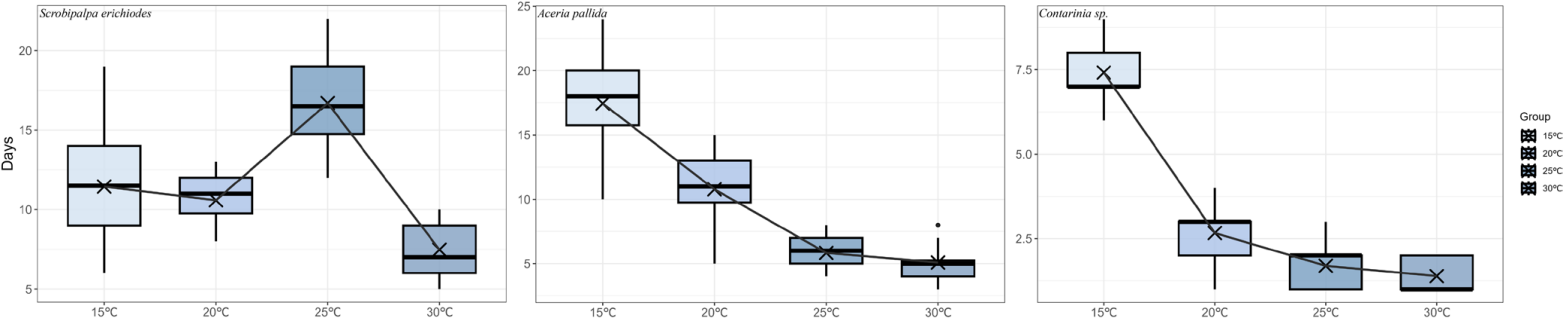
Effects of temperature on the adult longevity of the three gall inducers.

### 3.2 Composition of the Parasitoid Complex Associated With Gall Inducers

Notably, our results demonstrate high host specificity within this tritrophic network: with the exception of *C. pictus* (which exhibited a cross‐guild host shift to the gall mite *A. pallida* ), the dominant parasitoids were strictly exclusive to their respective host gall types, with zero species overlap recorded among the *S. erichiodes*, *A. pallida* , and *Contarinia* sp. parasitoid guilds.

Our study reveals that the three gall inducers on *Lycium ruthenicum* in northern Xinjiang support a diverse parasitoid community. A total of 13 parasitoid species belonging to 8 families were reared and identified (Table 5). Analysis of the community structure indicates that each gall inducer is regulated by distinct dominant parasitoids: *Pholetesor teresitergum* , *Cirrospilus pictus*, and *Pseudotorymus jaapiellae* were identified as the absolute dominant species associated with the *S. erichiodes*, *A. pallida* , and *Contarinia* sp. guilds, respectively.

**TABLE 5:** Community composition and dominance of parasitoids associated with the three gall inducers.

Pest	Natural enemy	New record	Community dominance (%)	Functional group dominance (%)
*Scrobipalpa erichiodes*	*Pholetesor teresitergum*	√	21.21	56.76
	*Mesopolobus* sp.	√	7.07	18.92
	*Agathidis* sp.	√	3.03	8.11
	*Telenomus* sp.	√	3.03	8.11
*Aceria pallida*	*Cirrospilus pictus*	×	66.39	66.39
	*Neochrysocharis formosus*	√	12.99	12.99
	*Aprostocetus* sp.	√	4.12	4.12
	*Trichogramma pintoi*	√	1.86	1.86
	*Aphelinoidea anatolica*	√	8.87	8.87
	*Lymaenon karlik*	√	2.12	2.12
	*Polynema haloxyloniae*	√	3.70	3.70
*Contarinia* sp.	*Pseudotorymus jaapiellae*	√	9.36	62.50
	*Platygaster* sp.	√	5.61	37.50

Notably, this study significantly expands the known host ranges of these parasitoids. Of the 13 species recovered, 12 represent new host records, with *Cirrospilus pictus* being the only previously recorded association. Specifically, *Pholetesor teresitergum* , *Mesopolobus* sp., *Agathidis* sp., and *Telenomus* sp. are reported as parasitoids of *S. erichiodes* for the first time. Furthermore, *Neochrysocharis formosus*, *Aprostocetus* sp., *Trichogramma pintoi*, *Aphelinoidea anatolica*, *Lymaenon karlik*, and *Polynema haloxyloniae* were newly recorded parasitizing *A. pallida* , while *Pseudotorymus jaapiellae* and *Platygaster* sp. represent new host records for *Contarinia* sp. These findings highlight the previously underestimated complexity of the plant‐herbivore‐parasitoid tritrophic interactions within this arid desert ecosystem.

### 3.3 Morphological Redescriptions of Dominant Parasitoids

To confirm the specific identities of the natural enemies associated with the gall inducers, detailed morphological examinations were conducted. This section provides brief diagnostic summaries of the three dominant parasitoids. The comprehensive full‐body morphological redescriptions of these three dominant species, along with the morphological descriptions and illustrations of the remaining 10 non‐dominant parasitoid species, are all provided in Appendix S1.

#### 3.3.1 *Pholetesor teresitergum* Liu & Chen, 2016 (Figure 12)

**FIGURE 12:**
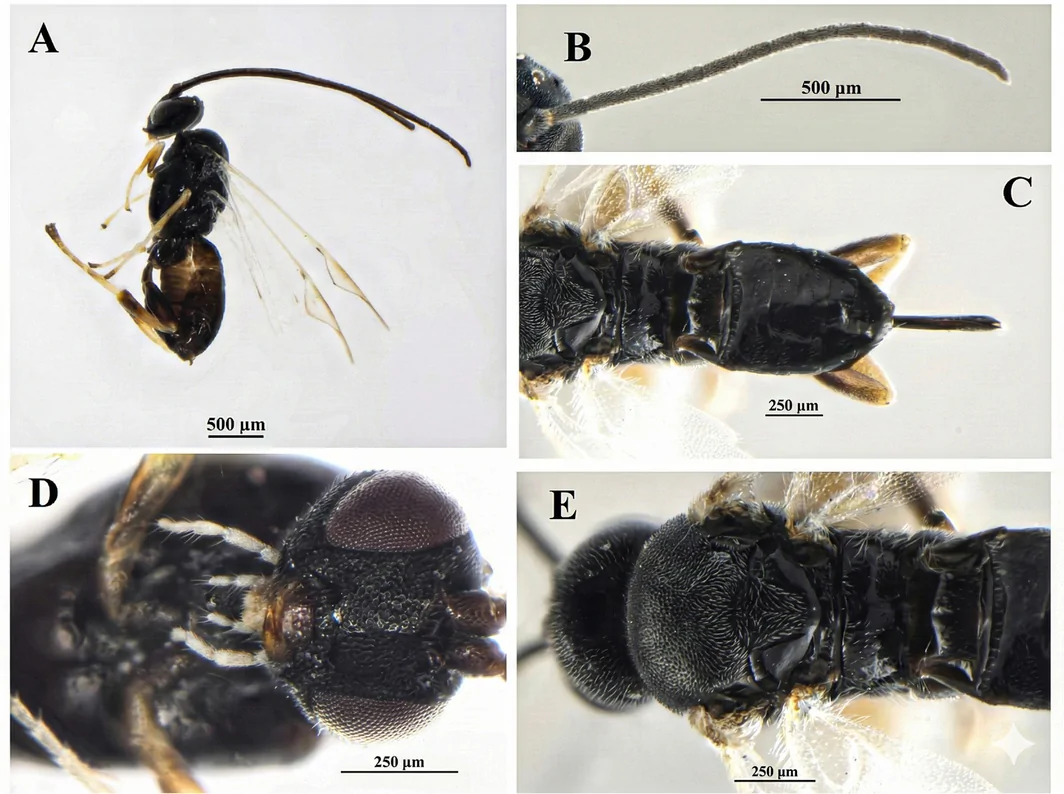
Diagnostic morphological characteristics of *Pholetesor teresitergum* . (A) Adult (lateral view); (B) Antenna; (C) Abdomen; (D) Head (frontal view); (E) Thorax.

This species is morphologically characterized by its black head and mesosoma, brownish‐yellow metasoma, and densely setose, 16‐segmented antennae. A key diagnostic wing feature is the absence of vein r‐m in the forewing, resulting in an open areolet. For full morphological details, refer to Appendix S1.

Host: Larvae of *Scrobipalpa erichiodes* (Lepidoptera: Gelechiidae).

Distribution: China (Xinjiang, Zhejiang).

#### 3.3.2 *Cirrospilus pictus* Nees, 1834 (Figure 13)

**FIGURE 13:**
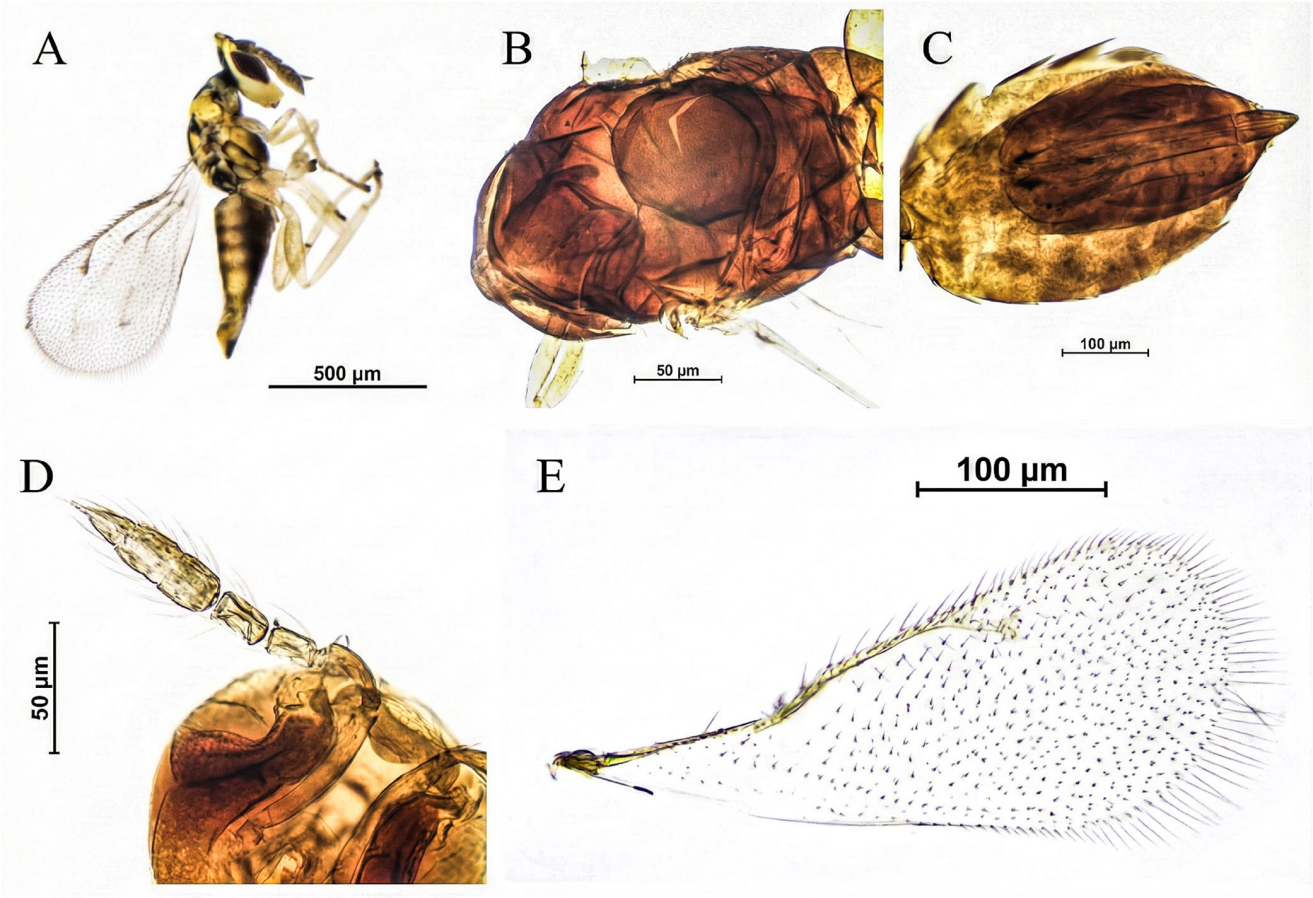
Diagnostic morphological characteristics of *Cirrospilus pictus*. (A) Adult (dorsal view); (B) Thorax; (C) Abdomen; (D) Antenna; (E) Forewing.

This eulophid wasp is easily distinguished by its yellowish‐brown body and a characteristic circular dark spot present at the junction of the pronotum and the mid‐lobe of the mesoscutum. The tarsi are distinctly 4‐segmented. For full morphological details, refer to Appendix S1.

Hosts: Generally primary parasitoids of various leaf‐mining insects; uniquely recorded in this study as a dominant parasitoid of the gall mite *A. pallida* .

Distribution: Widespread globally, including China (Xinjiang).

#### 3.3.3 *Pseudotorymus jaapiellae* Yang & Chen, 1987 (Figure 14)

**FIGURE 14:**
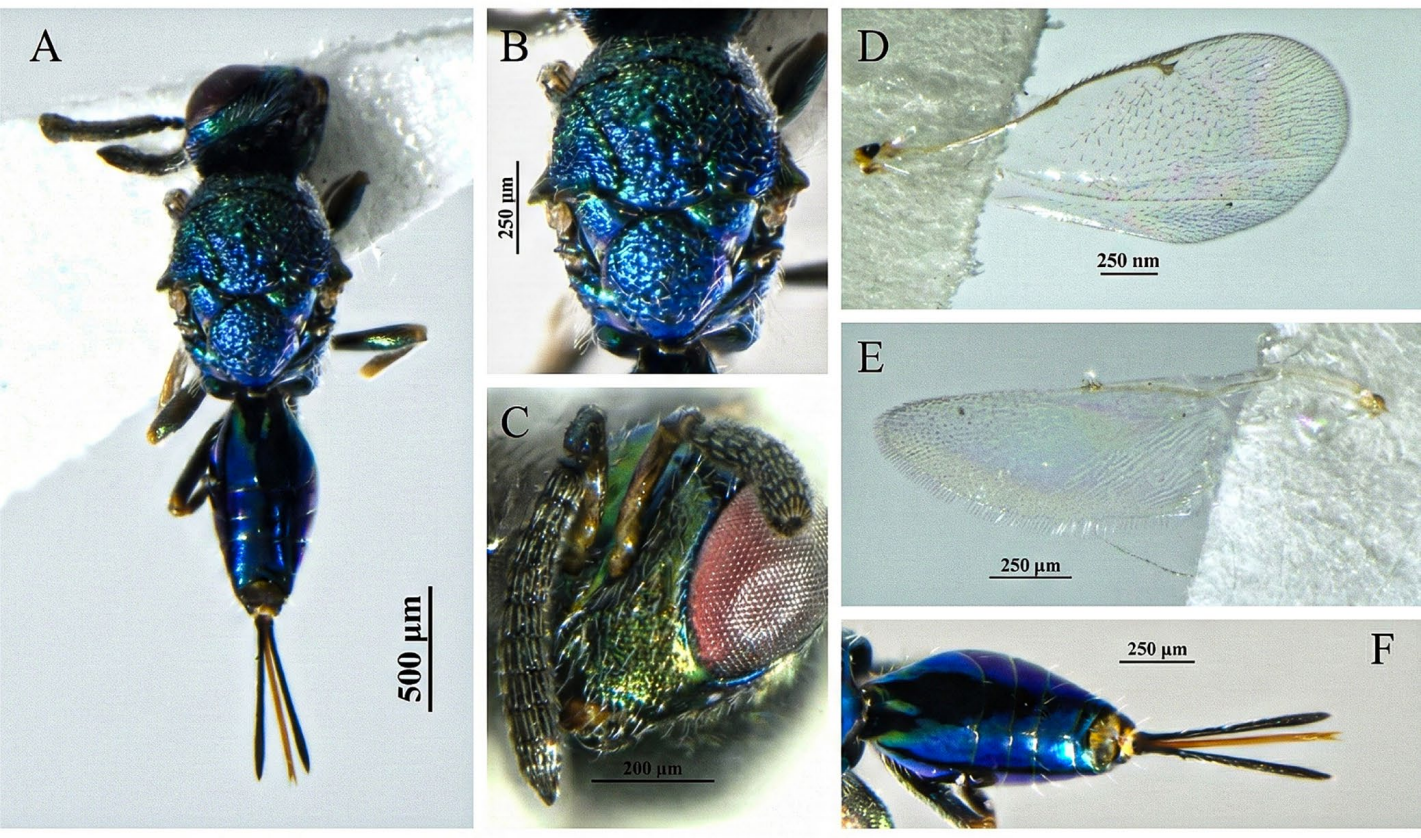
Diagnostic morphological characteristics of *Pseudotorymus jaapiellae*. (A) Adult (dorsal view); (B) Thorax; (C) Antenna; (D) Forewing; (E) Hindwing; (F) Abdomen.

This species exhibits a distinct bluish‐green body with a strong metallic luster, red compound eyes, and 13‐segmented antennae inserted in the middle of the face. The females possess a long ovipositor sheath approximately 1.2 × the length of the gaster. For full morphological details, refer to Appendix S1.

Hosts: *Gephyraulus lyciicola* (Diptera: Cecidomyiidae) and *Contarinia* sp. (Diptera: Cecidomyiidae).

Distribution: China (Ningxia, Xinjiang).

### 3.4 Phenology and Emergence Dynamics of Parasitoids

Statistical analysis of the emergence timing of parasitoids associated with different gall types revealed distinct seasonal patterns tailored to their specific host guilds (Figure 15). For the parasitoids associated with the swollen club‐shaped galls induced by *S. erichiodes*, the emergence dynamics exhibited a bimodal pattern with the first peak occurring in early‐to‐mid June and a second peak from mid‐August to early September; notably, temporal niche differentiation was observed among species, as the dominant parasitoid *P. teresitergum* emerged primarily in August and *Mesopolobus* sp. peaked from late August to mid‐September, whereas other associated parasitoids emerged mainly from late May to mid‐June. In contrast, adult emergence in the leaf gall guild associated with *A. pallida* was continuous, spanning from May to October; the dominant species *C. pictus* exhibited peak emergence in July, *Neochrysocharis formosus* emerged primarily in August, *Aprostocetus* sp. displayed two emergence peaks in June and August, and the remaining occasional species emerged predominantly in early June. Finally, the peak parasitism period for the flower bud gall guild associated with *Contarinia* sp. occurred from July to September, with the dominant parasitoid *P. jaapiellae* emerging mainly in late July and *Platygaster* sp. showing a distinct emergence peak in early September.

**FIGURE 15:**
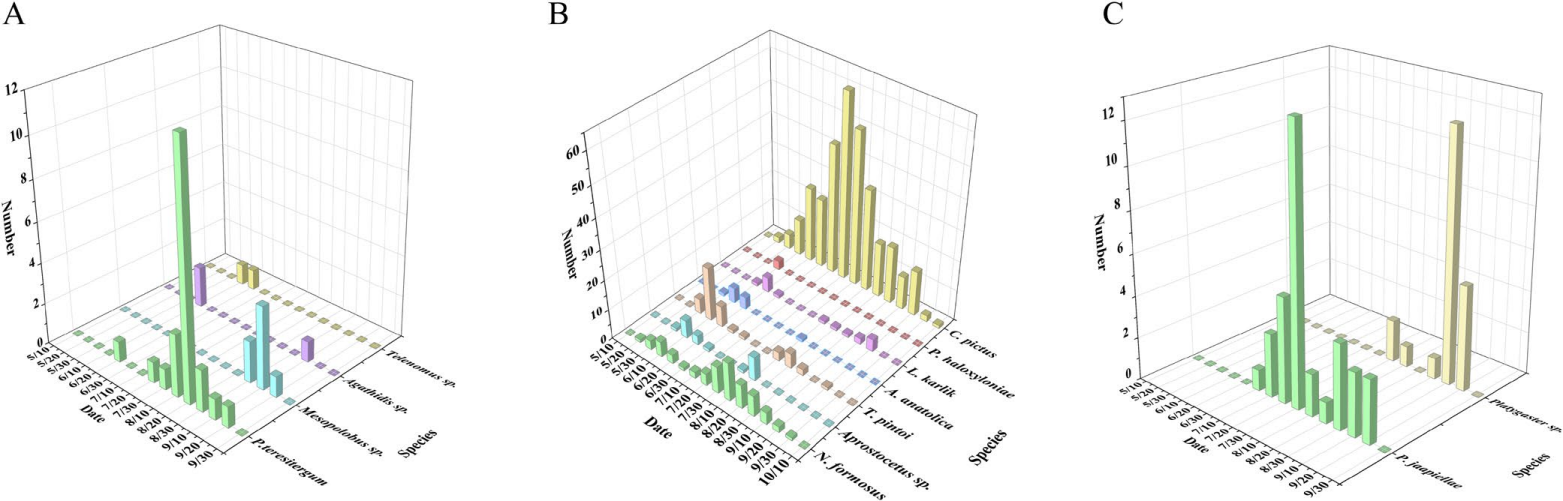
Seasonal emergence dynamics of parasitoids associated with the three gall inducers on *Lycium ruthenicum*. (A) Parasitic wasps associated with *S. erichiodes*; (B) Parasitic wasps associated with *A. pallida* ; (C) Parasitic wasps associated with *Contarinia* sp.

### 3.5 Parasitic Characteristics of Dominant Parasitoids

#### 3.5.1 Biological Characteristics of *Pholetesor teresitergum*

The morphological characteristics of *Pholetesor teresitergum* vary distinctively across its developmental stages. The larvae (Figure 16A,B) are initially pale yellow, transitioning to grayish‐white in later stages, and are characterized by a smooth body surface with progressively distinct segmentation, while the pre‐pupa (Figure 16C,D) appears pale yellow with a clearly differentiating head, thorax, and abdomen. Ecologically, *P. teresitergum* functions as a solitary ectoparasitoid attacking the larval stage of *Scrobipalpa erichiodes*. Given that the swollen club‐shaped galls are unilocular, the female wasp locates the host by antennal drumming on the gall surface and subsequently inserts her ovipositor to deposit eggs within the gall chamber. Upon hatching, the parasitoid larva attaches to the surface of the host larva and feeds on its haemolymph; as feeding progresses, the host larva becomes desiccated and eventually dies. Upon completing development, the adult wasp (Figure 16E) uses its mandibles to chew a circular emergence hole (Figure 16F) in the gall wall to exit. Furthermore, temperature significantly influenced the longevity of adult *P. teresitergum* . Within the experimental range of 15°C–30°C, a general trend of decreasing longevity with increasing temperature was observed (Figure 17). Adults lived longest under cooler conditions (15°C), while extreme thermal stress at 30°C reduced their lifespan by approximately half. Kaplan–Meier survival analysis confirmed that temperature significantly constrained the overall survival probability distribution of adult *P. teresitergum* (Log‐rank test: *χ* ^2^ = 8.82, *p* < 0.05, Figure S1).

**FIGURE 16:**
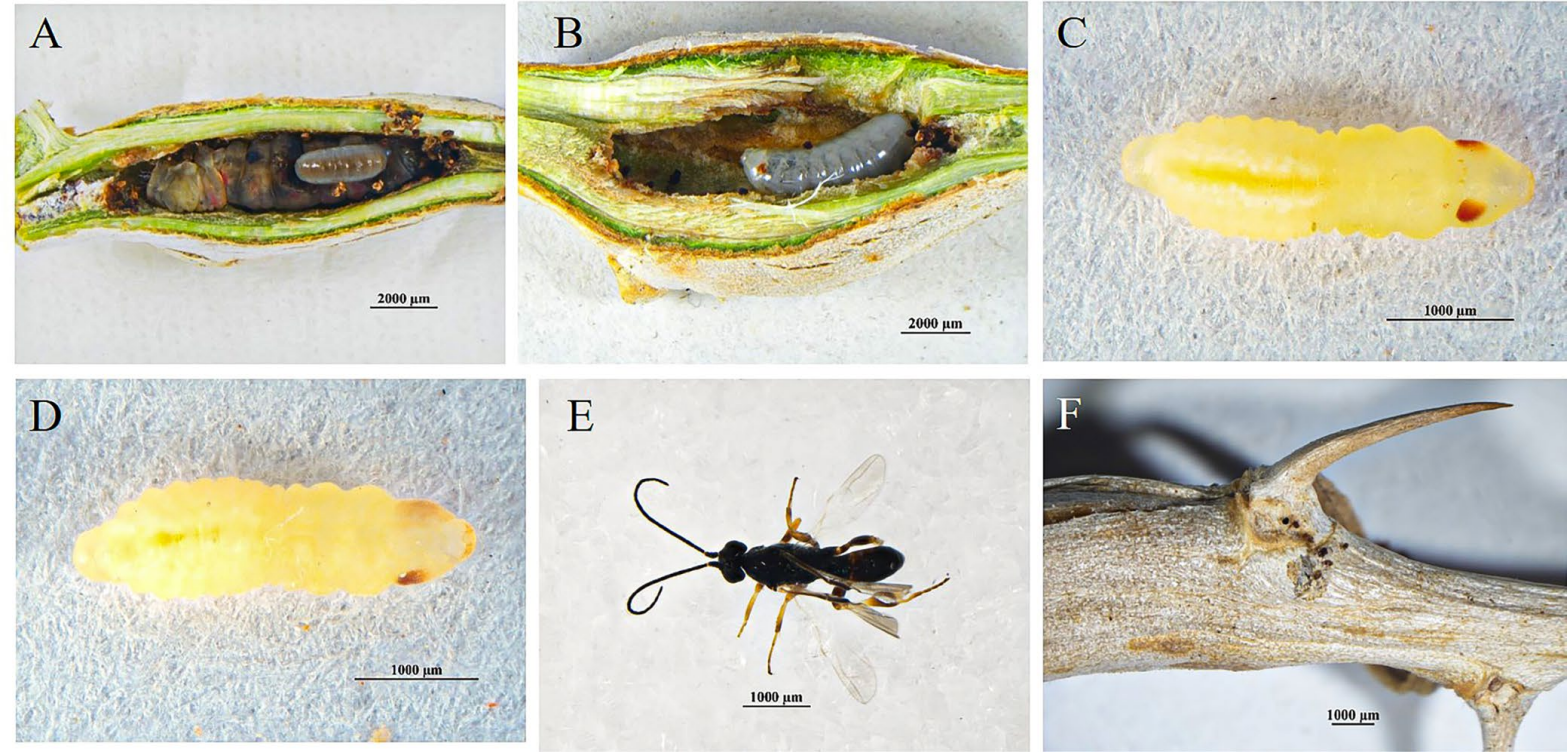
Developmental stages of *Pholetesor teresitergum* . (A, B) Larvae; (C, D) Pre‐pupa and pupa; (E) Adult; (F) Emergence hole on host gall.

**FIGURE 17:**
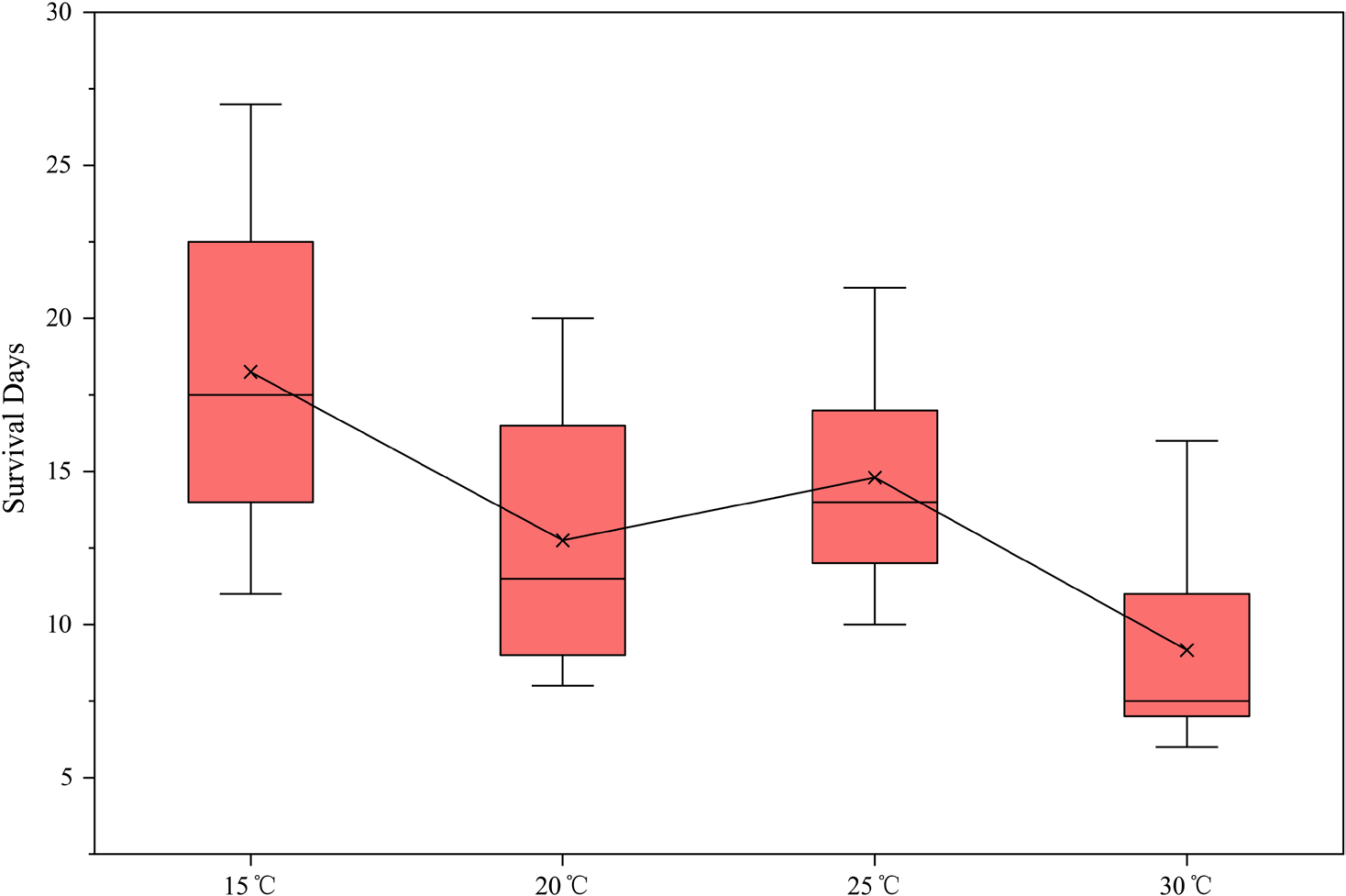
Adult longevity of *Pholetesor teresitergum* under different constant temperatures.

#### 3.5.2 Biological Characteristics of *Cirrospilus pictus*

The morphological features of *Cirrospilus pictus* vary distinctly during development. The larvae (Figure 18A,B) are initially creamy white, transitioning to grayish‐white in later stages with a brown mid‐body region; as development progresses into the pre‐pupa stage (Figure 18C), the body coloration darkens, and segmentation becomes increasingly distinct. The pupa (Figure 18D,E) is exarate and brownish‐black, with clearly differentiated head, thorax, and abdomen. Ecologically, *C. pictus* acts primarily as an ectoparasitoid of *A. pallida* . The adult female (Figure 18F) locates suitable hosts using antennal sensilla and deposits eggs within the chamber of the leaf gall. Upon hatching, the larvae exhibit a mixed feeding strategy, consuming both the gall mites and the parenchyma tissue within the gall. This feeding activity leads to the desiccation and chlorosis of the gall tissue, thereby deteriorating the microenvironment within the chamber. Upon completing development, adults emerge through fissures in the gall. Furthermore, temperature exerted a significant negative influence on the longevity of adult *C. pictus* . Within the experimental range of 15°C–30°C, a progressive decline in average lifespan was observed with increasing temperature (Figure 19). Adults exhibited their longest lifespan at 15°C, followed by a sharp and progressive decline as temperatures rose, reaching a minimum at 30°C. Kaplan–Meier survival analysis demonstrated that temperature significantly impacted the overall survival probability distribution of adult *C. pictus* (Log‐rank test: *χ* ^2^ = 21.32, *p* < 0.001, Figure S1).

**FIGURE 18:**
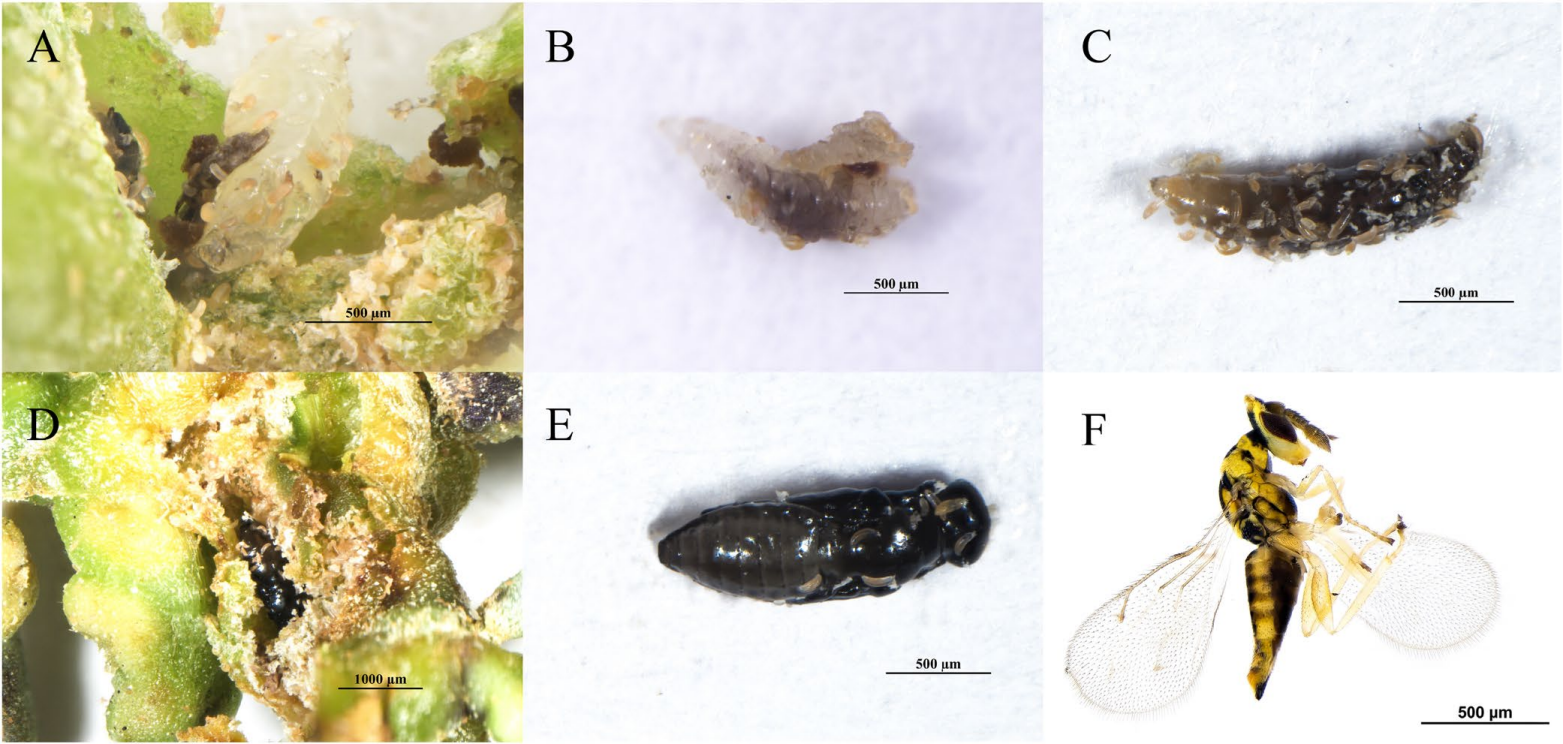
Developmental stages of *Cirrospilus pictus*. (A, B) Larval stage; (C) Pre‐pupa; (D, E) Pupal stage; (F) Adult.

**FIGURE 19:**
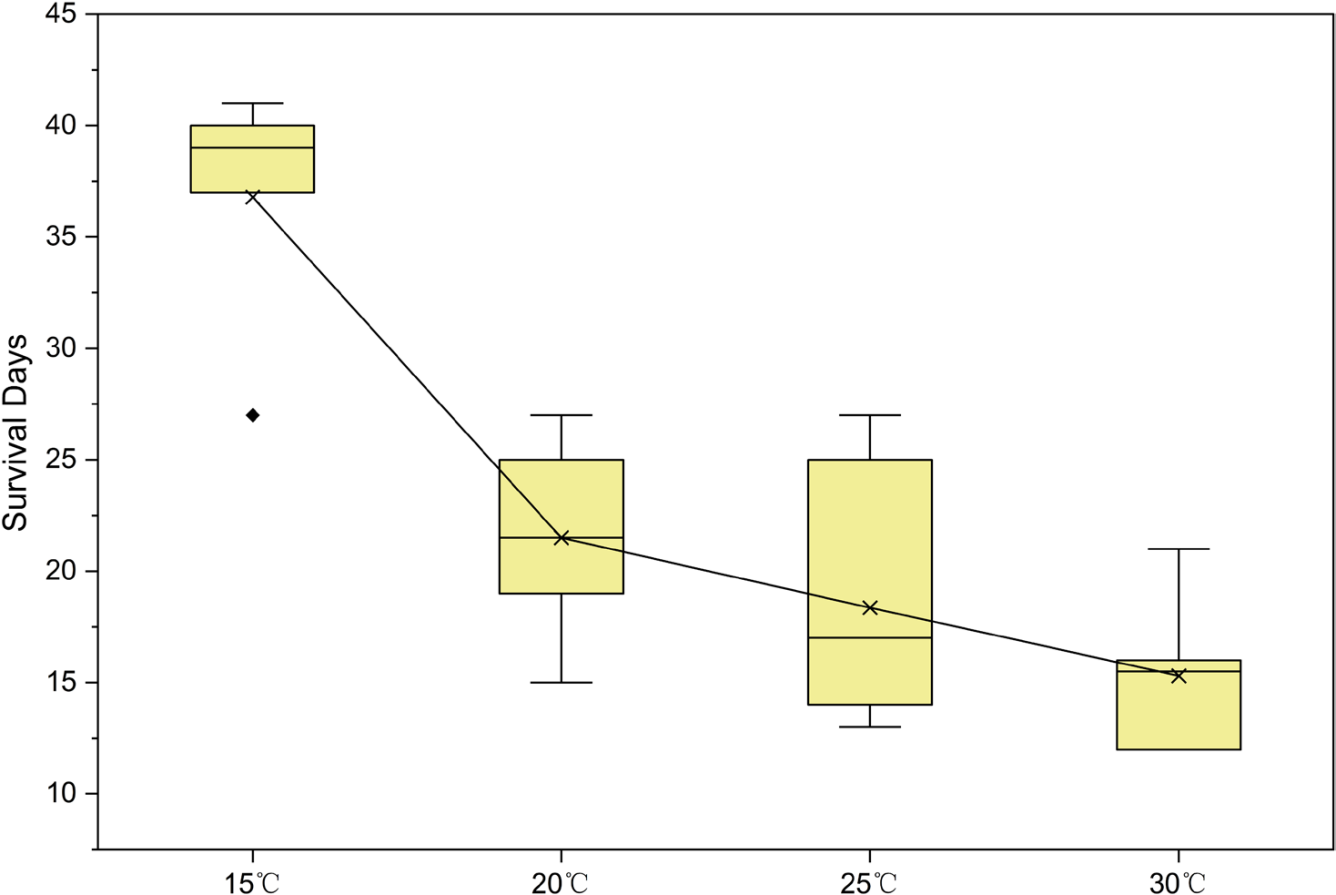
Adult longevity of *Cirrospilus pictus* under different constant temperatures.

#### 3.5.3 Biological Characteristics of *Pseudotorymus jaapiellae*

The morphological characteristics of *Pseudotorymus jaapiellae* vary distinctly throughout its development. The larvae (Figure 20A,B) appear pale yellowish‐white initially, turning white in later stages, and are characterized by a smooth, clearly segmented body with yellow‐green contents visible within the alimentary canal. The pupa (Figure 20C,D) is a yellowish‐brown exarate pupa, showing progressive differentiation of the head, thorax, and abdomen. Ecologically, *P. jaapiellae* functions as a solitary ectoparasitoid of *Contarinia* sp. larvae. The female wasp inserts her ovipositor into the flower bud gall to deposit eggs; upon hatching, the larva attaches to the host and feeds on its haemolymph. Notably, the parasitoid was observed to paralyze multiple host larvae prior to feeding. Rearing experiments also revealed that both the parasitoid and the host adult could emerge from the same gall, indicating that the parasitoid does not necessarily eliminate all host larvae within a multi‐chambered or multi‐host gall. Upon completing development, the adult wasp (Figure 20E) uses its mandibles to chew an emergence hole (Figure 20F) in the gall wall and exits after its cuticle has fully hardened. Temperature significantly affected the longevity of adult *P. jaapiellae*, with an inverse relationship observed within the 15°C–30°C range (Figure 21). Adults exhibited their maximum lifespan at 15°C. Similar to the other evaluated parasitoids, their longevity was severely restricted by elevated temperatures, dropping to a minimum at 30°C. Kaplan–Meier survival analysis demonstrated that thermal gradients significantly affected the survival probability distribution of adult *P. jaapiellae* (Log‐rank test: *χ* ^2^ = 11.30, *p* < 0.05, Figure S1).

**FIGURE 20:**
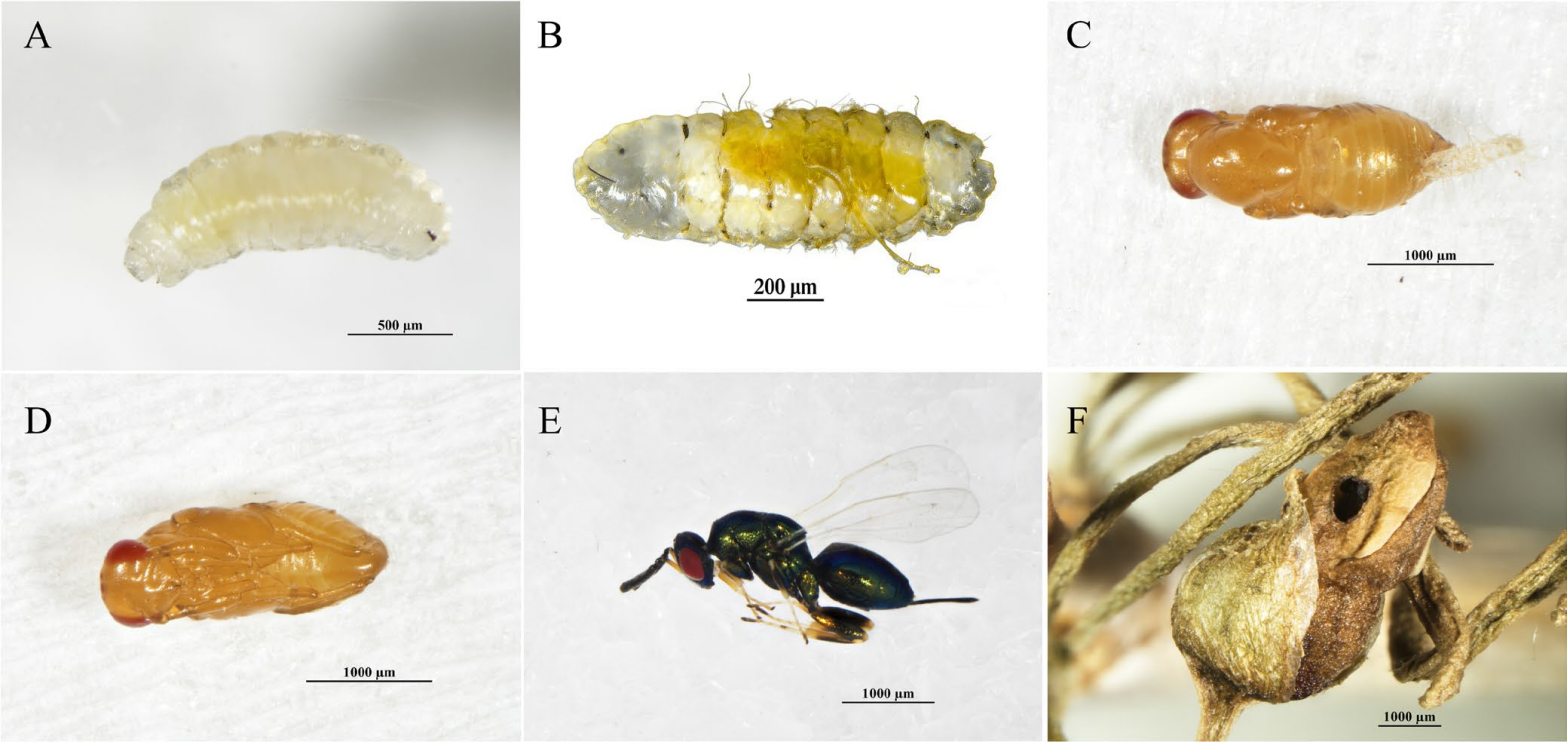
Developmental stages of *Pseudotorymus jaapiellae*. (A, B) Larva; (C, D) Pupa; (E) Adult; (F) Emergence hole on host gall.

**FIGURE 21:**
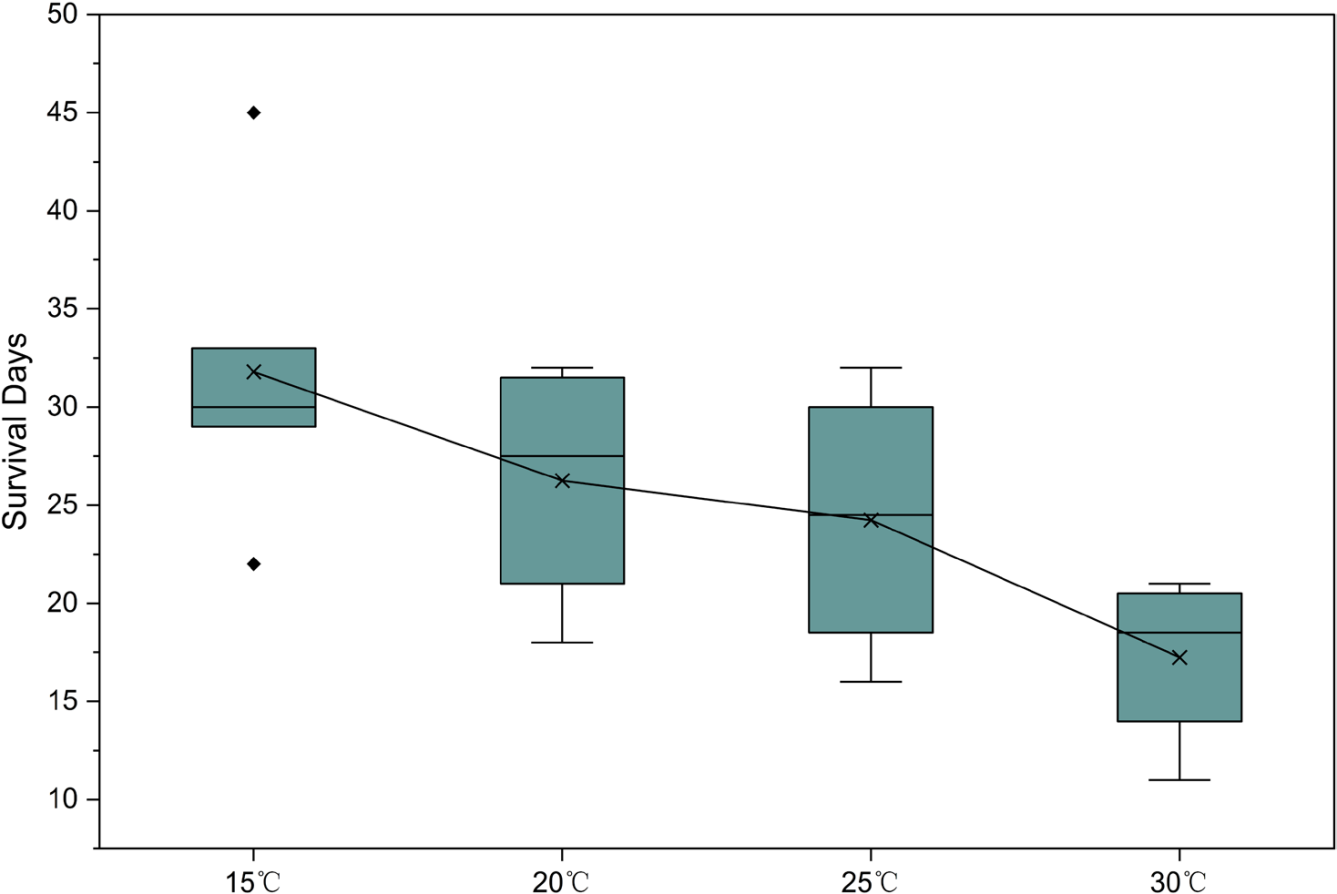
Adult longevity of *Pseudotorymus jaapiellae* under different constant temperatures.

## 4 Discussion

While *L. ruthenicum* is widely recognized as a keystone shrub maintaining the stability of arid desert ecosystems in northwest China (Dhar et al. 2011; Peng et al. 2014), previous research has predominantly focused on its physiological drought resilience, germplasm resources, and economic or medicinal value (Wang, Li, et al. 2018; Liu et al. 2020; Zhou, Wu, et al. 2022; Rehman et al. 2024). In contrast, there remains a critical paucity of comprehensive information regarding the basic ecology of the plant‐herbivore‐parasitoid networks associated with this species (Wu et al. 2022; Li et al. 2024). This study represents the first systematic elucidation of the tritrophic interactions centered on *L. ruthenicum* in northern Xinjiang. By definitively identifying three distinct gall types—induced by *A. pallida* , *Contarinia* sp., and the globally novel stem‐galler *S. erichiodes*—and uncovering a highly complex community of 13 associated parasitoid species, our findings bridge a significant knowledge gap in desert ecological networks and provide a critical baseline for top‐down biological control strategies in arid environments.

### 4.1 Niche Differentiation and Multidimensional Validation of the “Nutrition Hypothesis”

We report, for the first time globally, the gall‐inducing capability of a species within the genus *Scrobipalpa*, specifically *S. erichiodes*, which induces swollen club‐shaped galls on the stems of *L. ruthenicum*. Regarding the galling mechanism, we observed that larvae induce the hyperplasia of pith cells and their differentiation into nutritive tissues. This finding provides strong empirical support for the “Nutrition Hypothesis” (Price et al. 1986; Rezende et al. 2019). Guedes et al. 2023 noted that gall‐inducing Lepidoptera can reprogram plant tissue differentiation to generate lipid‐ and protein‐rich nutritive cells for consumption. Our results align closely with this observation and corroborate Miller's findings on stem galls induced by the con‐familial genus *Gnorimoschema*, where larvae establish a “nutrient sink” by altering host carbon allocation (Miller 2000). Tooker et al. further demonstrated that stem galls not only alter primary metabolism but also significantly enhance secondary metabolite levels (Tooker and De Moraes 2007). Collectively, these findings suggest that inducing tissue differentiation to construct a high‐nutrient microenvironment is a convergent evolutionary strategy adopted by gall‐inducing Gelechiidae across different taxa.

### 4.2 Physical Constraints of Lignified Stems on Gall Phenotypic Plasticity

It is generally accepted that gall size is positively correlated with the body size of the inducing insect (Egan et al. 2013). However, our study revealed that this correlation weakened during the late developmental stages of the *S. erichiodes* galls, deviating from a typical linear growth model. This discrepancy can be attributed to the physical constraints imposed by the host plant organs. Comparative morphological studies have shown that stem‐derived galls exhibit lower phenotypic plasticity than leaf galls due to the constraints of vascular bundles and the lignification process (Bessho‐Uehara et al. 2025). The rapid lignification and high hardness of *L. ruthenicum* branches likely limit radial expansion during the mid‐developmental stage of the gall, resulting in external dimensions that do not fully reflect the growth status of the internal larvae. This underscores that when studying stem galls on woody plants, internal insect stages cannot be predicted solely based on external morphology; rather, an integrated analysis incorporating plant anatomical features is required.

### 4.3 Overwintering Strategies and Environmental Adaptation of Gall Mites

We found that *Aceria pallida* primarily overwinters in branch crevices and axillary buds, exhibiting high fecundity closely linked to its strict host specificity (Castagnoli et al. 2010; Petanović and Kielkiewicz 2010). Notably, Wu et al. (2022) reported that this mite can utilize the psyllid *Bactericera gobica* as a vector (phoresy) for dispersal and overwintering, a strategy that significantly enhances survival in harsh environments. Combined with the high degree of generation overlap observed in this study, it suggests that *A. pallida* has evolved diverse adaptive mechanisms—through microhabitat selection and potential vector‐mediated dispersal—which explains its perennial population outbreaks in desert ecosystems.

### 4.4 Host Shifting and Cross‐Guild Parasitism in the Parasitoid Community

This study elucidates a complex tritrophic network within the natural enemy community. The identification of *P. teresitergum* as the dominant parasitoid of *S. erichiodes* aligns with the established pattern of Braconidae primarily parasitizing lepidopteran larvae (Fernandez‐Triana et al. 2016; Ridgway and Mahr 1990). The discovery of *C. pictus* as the dominant parasitoid of the gall mite *A. pallida* represents a remarkable trophic expansion. Historically, *C. pictus* has been classically documented as a primary ectoparasitoid attacking specifically leaf‐mining insects across Lepidoptera, Coleoptera, and Diptera (Zhu et al. 2002). Its success in exploiting gall‐inducing mites may be attributed to the structural parallels between leaf mines and leaf galls; both provide a concealed, shallow endophytic microhabitat that shields the parasitoid larvae while remaining physically accessible to the ovipositor of the adult female wasp (Stone and Schönrogge 2003). Furthermore, in the harsh, resource‐limited arid environments of northern Xinjiang, parasitoids are often subjected to strong selective pressures that favor opportunistic foraging strategies and host plasticity (Stireman III et al. 2005). This extraordinary adaptability not only challenges conventional host‐range paradigms for Eulophidae wasps but also highlights the untapped potential of *C. pictus* as a highly versatile biological control agent against diverse gall‐forming pests in desert agriculture. Additionally, the dominant parasitoid of *Contarinia* sp. was identified as *Pseudotorymus jaapiellae* (Torymidae). Given that Copland highlighted Torymidae as primary natural enemies of gall midges, our results further confirm the pivotal role of this family in the biological control of cecidomyiids (Copland and King 1972).

### 4.5 Constraints of Thermal Stress on Parasitoid Longevity

Temperature is a critical abiotic factor determining insect population dynamics (Zhao et al. 2021; Gopko et al. 2020). Our study demonstrated a significant decline in the longevity of adults of all three dominant parasitoids as temperatures increased from 15°C to 30°C. This result is consistent with findings by Picciau et al. and Wang et al. (Picciau et al. 2017; Wang, Lao, et al. 2018) regarding thermal effects on parasitoids, and is further supported by Dhillon and Sharma (Dhillon and Sharma 2009) and Xia et al. (Xia et al. 2025), who noted that high temperatures shorten parasitoid lifespan. In northern Xinjiang, where summer is characterized by large diurnal temperature variations and frequent extreme heat, such thermal stress may significantly compress the effective foraging window for parasitoids, representing a bottleneck that limits their natural control efficiency. Therefore, future biological control strategies should prioritize releasing natural enemies during the milder early summer period or selecting strains with higher thermal tolerance to adapt to the desert climate.

In several cases, divergences between the thermal preferences of the host and those of the parasitoid lead to a disruption of the temporal or geographical synchronization, increasing the risk of host outbreaks (Hance et al. 2007). Crucially, our thermal performance comparisons reveal a distinct thermal sensitivity mismatch between gall‐inducing herbivores and their associated parasitoid communities in arid desert environments. While gall‐inducing herbivores such as *S. erichiodes* and *A. pallida* maintain functional longevity across broader thermal windows (peaking or plateauing around 25°C), the adult lifespans of primary parasitoids ( *P. teresitergum* , *C. pictus* , and *P. jaapiellae*) experience severe compression under elevated temperatures (30°C). This physiological disparity likely stems from the physical buffering provided by gall structures. Gall tissues act as microclimatic refugia that protect internal‐feeding larvae from extreme ambient heat and desiccation (Price et al. 1986; Stone and Schönrogge 2003; Pincebourde and Casas 2019). In contrast, free‐living adult parasitoids are directly exposed to the hostile, low‐humidity microclimate of northern Xinjiang desert ecosystems, accelerating energy consumption and desiccation‐induced mortality (Chown and Nicolson 2004; Jeffs and Lewis 2013).

From a tri‐trophic interaction perspective, this thermal mismatch represents a major bottleneck for natural biological control in warming desert ecosystems (Tylianakis et al. 2008). As summer temperatures in arid zones frequently exceed 30°C, the drastic reduction in adult parasitoid longevity inevitably narrows their temporal window for host searching and oviposition, potentially resulting in phenological desynchronization between host vulnerability and parasitoid attack (van Asch and Visser 2007; Forrest 2016). Consequently, reliance solely on natural parasitoid suppression may be insufficient during peak midsummer heat waves. Future integrated pest management (IPM) strategies for *L. ruthenicum* plantations should incorporate microclimate manipulation—such as canopy shade management or strategic micro‐irrigation—to mitigate ambient heat stress, prolong parasitoid field survival, and restore top‐down regulation in arid ecosystems (Landis et al. 2000; Gurr et al. 2016).

### 4.6 Limitations of Constant Temperature Experiments and Potential Impacts of Fluctuating Thermal Regimes

Our laboratory assays revealed a significant negative correlation between constant temperature (15°C–30°C) and the longevity of dominant parasitoids. However, it is imperative to acknowledge that arid desert ecosystems in northern Xinjiang are characterized by pronounced Diurnal Temperature Variations (DTV). In natural habitats, insects are subjected to periodic thermal fluctuations rather than the steady‐state thermal stress maintained under controlled laboratory conditions.

Existing ecological theory suggests that the impact of fluctuating temperatures on insect physiological performance often follows Jensen's Inequality. Specifically, within the non‐linear regions of Thermal Performance Curves (TPCs), performance under fluctuating conditions may deviate significantly from that observed at a constant temperature equal to the mean. For the dominant parasitoids identified in this study— *P. teresitergum* , *C. pictus* , and *P. jaapiellae*—lower nocturnal temperatures may provide a critical window for “thermal recovery,” potentially mitigating physiological damage incurred during extreme diurnal heat peaks.

Consequently, while our constant‐temperature data establish a valuable baseline for assessing thermal thresholds, relying solely on such regimes may overestimate the negative impacts of extreme heat on parasitoid populations in the field. Future research should incorporate fluctuating thermal regimes that simulate natural desert patterns to more accurately predict the long‐term stability of tritrophic interactions and community structure under changing climate conditions.

## 5 Conclusions

This study provides the first systematic elucidation of the biological characteristics and tritrophic interactions of the three primary gall inducers infesting *Lycium ruthenicum* in northern Xinjiang. We definitively confirm *Scrobipalpa erichiodes* as the first species within the genus *Scrobipalpa* described globally to possess gall‐inducing capabilities. Within the identified parasitoid community of 13 species, *Pholetesor teresitergum* , *Cirrospilus pictus*, and *Pseudotorymus jaapiellae* were determined to be the dominant natural enemies of the three respective gall inducers. Most notably, we demonstrate that *C. pictus* efficiently parasitizes *A. pallida* (with a dominance of 66.39%). This finding challenges the conventional understanding that this wasp attacks only leaf‐mining insects and represents a significant novel trophic association. Furthermore, our results reveal that high temperature (30°C) serves as a critical abiotic constraint, significantly reducing the adult longevity and potential control efficiency of these dominant parasitoids. Collectively, these findings not only fill critical gaps in the basic biology of *L. ruthenicum* gall inducers but also provide a theoretical foundation for selecting heat‐tolerant parasitoid strains and developing seasonal biological control strategies tailored to arid desert ecosystems.

## Author Contributions

**Gaopeng Yu:** conceptualization (equal), data curation (lead), formal analysis (lead), methodology (equal), software (equal), writing – original draft (equal), writing – review and editing (equal). **Yuhao Nie:** conceptualization (equal), investigation (equal), methodology (equal), software (equal), writing – review and editing (equal). **Caihong Gao:** conceptualization (equal), investigation (equal), software (equal), writing – original draft (equal). **Hongying Hu:** conceptualization (equal), funding acquisition (lead), methodology (equal), project administration (lead), supervision (lead), writing – review and editing (equal).

## Funding

This study was supported by The Third Xinjiang Integrated Scientific Expedition (grant no. 202106120032), a project supported by the Ministry of Science and Technology of the People’s Republic of China.

## Conflicts of Interest

The authors declare no conflicts of interest.

## Supporting information


**Figure S1:** Survival analysis curve of three gall inducers of *Lycium ruthenicum* and Their Dominant Parasitic Wasps.

**Figure S1.1:** Morphology characteristics of *Mesopolobus* sp.
**Figure S1.2:** Morphological characteristics of *Agathidis* sp.
**Figure S1.3:** Morphological characteristics of *Telenomus* sp.
**Figure S1.4:** Morphological characteristics of *Aprostocetus* sp.
**Figure S1.5:** Morphological characteristics of *Neochrysocharis formosus*.
**Figure S1.6:** Morphological characteristics of *Lymaenon karlik*.
**Figure S1.7:** Morphological characteristics of *Polynema haloxyloniae*.
**Figure S1.8:** Morphological characteristics of *Trichogramma pintoi*.
**Figure S1.9:** Morphological characteristics of *Aphelinoidea anatolica* Novicky.
**Figure S1.10:** Morphological characteristics of *Platygaster* sp.

## Data Availability

The data for this study are available on: https://doi.org/10.6084/m9.figshare.32594304.
