# Supplementary material for: Tritrophic Interactions and Parasitoid Community Structure of Gall‐Inducing Herbivores in Arid Desert Ecosystems of Northern Xinjiang, China
Source: Ecol Evol. 2026 Oct 8;16(10):e74408. doi: 10.1002/ece3.74408 (PMC13649611; doi:10.1002/ece3.74408)
Supplement: Supplementary file 1 [file ECE3-16-e74408-s001.doc]

**Parasitoid Complex of *Scrobipalpa erichiodes***

**(1) *Mesopolobus* sp.** **Male:** Body length 3.89–5.13 mm. Body blue-green with metallic luster. Antennae yellowish-brown, 13-segmented, antennal formula 11353, inserted on the lower ocular line. Compound eyes reddish-brown, ocelli yellowish-brown. Ratio of head width to mesosomal width 1.15:1. Mesosoma with reticulate sculpture; pronotum relatively narrow, anteriorly carinate, ratio of length to width 10.4:1; notauli incomplete. Median area of propodeum smooth, with a median carina. Tegulae yellow. Forewing length 2.56–3.43 mm, width 1.12–1.39 mm; ratio of submarginal vein : stigmal vein : postmarginal vein 402:274:243. Hindwing length 1.92–2.28 mm, width 0.51–0.58 mm. Venation pale yellow. Ratio of gaster length to width 2.18:1. Coxae brown, femora yellowish-brown, tibiae and tarsi pale yellow.

**Female:** Unknown.

**Material examined:** 1♂, County Road 312, Wujiaqu City, Xinjiang, N: 44°20’11.062”, E: 87°27’41.270”, H: 450.9 m, 26.viii.2023, reared from the swollen club-shaped galls on *Lycium ruthenicum*; 2♂, County Road 312, Wujiaqu City, Xinjiang, N: 44°20’11.062”, E: 87°27’41.270”, H: 450.9 m, 02.ix.2023, reared from the swollen club-shaped galls on *Lycium ruthenicum*.

**Host:** Larvae of *Scrobipalpa erichiodes* (Lepidoptera: Gelechiidae).

**Distribution:** China (Xinjiang).

Figure S1.1. Morphology characteristics of *Mesopolobus* sp.

A: body, dorsal view; B: thora; C: thorax, lateral view; D: antennae; E: fore wing; F: abdomen

**(2) *Agathis* sp.**

**Male:** Body length 3.19–4.18 mm. Body black. Antennae 22-segmented, brownish-black, densely pubescent, inserted below the middle line of the compound eyes. Head in anterior view narrowing ventrally from the dorsal side, subtriangular. Ratio of posterior ocellar line (POL) to ocular-ocellar line (OOL) 1:1.19. Head black, occipital carina absent. Mesosoma black, densely covered with fine pubescence, ratio of length to width 1.92–2.15:1; pronotum smooth; notauli complete; mesoscutum and scutellum sparsely punctate. Forewing length 2.38–2.72 mm, width 0.76–0.84 mm. Venation brown; marginal cell relatively narrow; ratio of the 1st section of the radius (vein r) to the 1st radiomedial crossvein (vein 2-SR) 1:1.21; ratio of pterostigma length to width 2.5:1. Propodeum punctate. Ratio of metasoma length to width 2.42–2.77:1; second and third metasomal tergites yellowish-brown, remainder black. Coxae and femora yellowish-brown, tibiae and tarsi yellow.

**Female:** Unknown.

**Material examined:** 2♂, County Road 312, Wujiaqu City, Xinjiang, N: 44°20’11.062”, E: 87°27’41.270”, H: 450.9 m, 26.vii.2023, reared from the swollen club-shaped galls on *Lycium ruthenicum*.

**Host:** Larvae of *Scrobipalpa erichiodes* (Lepidoptera: Gelechiidae).

**Distribution:** China (Xinjiang).

Figure S1.2 Morphological characteristics of *Agathidis* sp.

A: antennae; B: fore wing; C: hind wing; D: abdomen; E: thorax

**(3) *Telenomus* sp.**

**Male:** Body length 0.91–0.97 mm. Body black. Head width 1.16 times head length, 1.12 times mesosomal width. Antennae 12-segmented; ratio of scape length to width 5.53:1; third antennomere to apex with short black setae; frons with longitudinal striae medially; mandibles brown; occipital carina present. Pronotum relatively narrow, invisible in dorsal view; mesoscutum punctate, densely covered with white pubescence; scutellum with sparse setae. Forewing brown, length 0.95–1.03 mm, width 0.26–0.28 mm; stigmal vein length 1.56 times marginal vein length. Hindwing length 0.82–0.85 mm, width 0.11–0.14 mm, venation reduced.

Metasoma oval, length 1.57 times width; T1 (first metasomal tergite) with two setae on each lateral side; T1 and T2 with longitudinal costae basally, smooth apically. T3–T6 with setae apically. Legs dark brown, except trochanters, tibiae, and tarsi yellowish-brown.

**Female:** Unknown.

**Material examined:** 1♂, County Road 312, Wujiaqu City, Xinjiang, N: 44°20’11.062”, E: 87°27’41.270”, H: 450.9 m, 04.vi.2023, reared from the swollen club-shaped galls on *Lycium ruthenicum*; 1♂, County Road 312, Wujiaqu City, Xinjiang, N: 44°20’11.062”, E: 87°27’41.270”, H: 450.9 m, 13.vi.2023, reared from the swollen club-shaped galls on *Lycium ruthenicum*.

**Host:** Eggs of *Scrobipalpa erichiodes* (Lepidoptera: Gelechiidae).

**Distribution:** China (Xinjiang).

Figure S1.3 Morphological characteristics of *Telenomus* sp.

A: antennae; B: head, frontal view; C: abdomen; D: wing; E: thorax

**Parasitoid Complex of Aceria pallida**

**(1) *Aprostocetus* sp.**

**Female:** Body length 0.91–1.08 mm. Body black with green metallic luster. Compound eyes red; ocelli arranged in an obtuse triangle. Antennal scape and pedicel yellowish-brown; funicle and clava pale yellow; inserted below the middle of the face; funicle 3-segmented, clava 3-segmented. Ratio of mesosomal length to width 1.14–1.27:1; pronotum relatively short; notauli distinct; mesoscutum with longitudinal striations and two pairs of setae; scutellum with two pairs of setae. Wings hyaline, venation pale yellow; ratio of forewing length to width 2.05–2.23:1, with relatively short marginal setae; wing disc with brown setae; marginal vein 5.5 times as long as stigmal vein; postmarginal vein absent.

Metasoma oval, length 1.66 times width; ovipositor exserted beyond the apex of the metasoma. Legs yellow, except bases of femora and apices of tarsi brown; tarsi 4-segmented.

**Male:** Antennal funicle 4-segmented; body size slightly smaller than female.

**Material examined:** 1♀2♂, County Road 312, Wujiaqu City, Xinjiang, N: 44°20’11.062”, E: 87°27’41.270”, H: 450.9 m, 05.vi.2023, reared from leaf galls on *Lycium ruthenicum*; 1♀, County Road 312, Wujiaqu City, Xinjiang, N: 44°20’11.062”, E: 87°27’41.270”, H: 450.9 m, 08.vi.2023, reared from leaf galls on *Lycium ruthenicum*; 1♀, Shuanghe City, Xinjiang, N: 44°52’35.907”, E: 82°29’32.424”, H: 295.8 m, 30.vi.2023, reared from leaf galls on *Lycium ruthenicum*; 1♀1♂, Company 9, Regiment 103, Wujiaqu City, Xinjiang, N: 44°29’48.026”, E: 87°30’53.920”, H: 363.8 m, 20.vii.2023, reared from leaf galls on *Lycium ruthenicum*.

**Host:** *Aceria pallida* (Acari: Eriophyidae).

**Distribution:** China (Xinjiang).

Figure S1.4 Morphological characteristics of *Aprostocetus* sp.

A: body, lateral view; B: fore wing; C: antennae; D: thorax; E: abdomen

**(2) *Neochrysocharis formosus* Westwood, 1833**

**Female:** Body length 1.17–1.41 mm. Body green with metallic luster. Head with distinct sculpture. Antennae brown, with linear sensilla; funicle 2-segmented, clava 3-segmented; ratio of clava length to width 3.06–3.5:1; length ratio of respective segments 18:20:15. Frontal sutures "V"-shaped.

Compound eyes and ocelli red; ratio of posterior ocellar line (POL) to ocular-ocellar line (OOL) 2.43:1. Ratio of mesosomal length to width 1.34–1.55:1; pronotum relatively short; mesoscutum with 2 pairs of setae, notauli incomplete; ratio of scutellum length to width 1.03–1.11:1, with 1 pair of setae; propodeum sculptured, median carina absent. Wings hyaline, venation brown; ratio of forewing length to width 2.15–2.44:1; postmarginal vein length 0.8 times stigmal vein length. Metasoma length 1.95 times width. Legs pale yellow, except apices of tarsi brown; tarsi 4-segmented.

**Male:** Body size slightly smaller than female; morphological characteristics similar to female; antennal scape slightly wider than in female.

**Material examined:** 1♀, County Road 312, Wujiaqu City, Xinjiang, N: 44°20’11.062”, E: 87°27’41.270”, H: 450.9 m, 09.vi.2023, reared from leaf galls on *Lycium ruthenicum*; 1♀1♂, County Road 312, Wujiaqu City, Xinjiang, N: 44°20’11.062”, E: 87°27’41.270”, H: 450.9 m, 12.vi.2023, reared from leaf galls on *Lycium ruthenicum*; 1♀2♂, Shigao Section, Manas County, Changji, Xinjiang, N: 44°30’35.075”, E: 86°23’52.919”, H: 398.5 m, 27.vii.2023, reared from leaf galls on *Lycium ruthenicum*; 1♂, Company 9, Regiment 103, Wujiaqu City, Xinjiang, N: 44°29’48.026”, E: 87°30’53.920”, H: 363.8 m, 20.vii.2023, reared from leaf galls on *Lycium ruthenicum*.

**Host:** Primarily parasitizes Coleoptera, Diptera, and Lepidoptera. Primary parasitoid of *Aceria pallida*; gall-inducing insects in leaf galls of *Nitraria sibirica* .

**Distribution:** China (Beijing, Guangdong, Guangxi, Yunnan, Gansu, Xinjiang, Taiwan); Japan, Malaysia, Russia, Poland, Turkey, Egypt, UK, France, Germany, Hungary, Spain, Switzerland, Norway, USA, Canada, Mexico.

Figure S1.5 Morphological characteristics of *Neochrysocharis formosus*

A: body, dorsal view; B: wing; C: abdomen; D: thorax; E: head.

**(3) *Lymaenon karlik* Triapitsyn, 2013**

**Female:** Body length 0.58–0.81 mm. Dark brown. Antennae pale yellowish-brown except funicle and clava dark brown; inserted on the middle interocular line; female antennae 11-segmented, funicle 8-segmented. Pronotum 3-lobed; mesoscutum sculptured. Forewing broadly rounded, length 2.91–3.35 times width, disc densely setose; longest marginal setae 0.29–0.37 times wing width; postmarginal and stigmal veins absent.

Hindwing narrow and long, longest marginal setae 2.04–2.73 times wing width. Metasomal tergites 1 to 3 yellow, remainder brown; ovipositor extending from the second metasomal segment to the apex of the metasoma, length occupying 0.69–0.83 of the metasoma. Coxae, bases of femora, apices of tibiae and tarsi brown, remainder yellow; tarsi 5-segmented.

**Male:** Body size slightly smaller than female; antennal flagellum 11-segmented.

**Material examined:** 2♀1♂, County Road 312, Wujiaqu City, Xinjiang, N: 44°20’11.062”, E: 87°27’41.270”, H: 450.9 m, 03.vi.2023, reared from leaf galls on *Lycium ruthenicum*; 1♀1♂, County Road 312, Wujiaqu City, Xinjiang, N: 44°20’11.062”, E: 87°27’41.270”, H: 450.9 m, 05.vi.2023, reared from leaf galls on *Lycium ruthenicum*; 1♀, Shigao Section, Manas County, Changji, Xinjiang, N: 44°30’35.075”, E: 86°23’52.919”, H: 398.5 m, 04.vi.2023, reared from leaf galls on *Lycium ruthenicum*.

**Host:** *Aceria pallida* (Acari: Eriophyidae).

**Distribution:** China (Beijing, Xinjiang); Russia, South Korea.

Figure S1.6 Morphological characteristics of *Lymaenon karlik*

A: body, lateral view; B: fore wing; C: female antennae; D: head; E: male antennae F: thorax; G: hind wing

**(4) *Polynema haloxyloniae***

**Female:** Body length 0.39–0.62 mm, brown. Compound eyes red. Antennae pale yellowish-brown; female antennae 9-segmented, funicle 6-segmented, clava 1-segmented; ratio of length to width for scape : pedicel : funicular segments (F1–F6) : clava is 71 : 55 : (23 : 39 : 39 : 29 : 28 : 43) : 11; intertorular depression absent. Pronotum and mesoscutum sculptured; scutellum longer than mesoscutum.

Forewing relatively narrow and long, ratio of length to width 4.3–4.76:1; venation relatively short; wing disc setose; longest marginal setae 0.24–0.35 times wing length; postmarginal and stigmal veins absent. Hindwing longest marginal setae 0.17–0.29 times wing length. Metasoma with a long petiole; ovipositor extending from the second metasomal segment to the apex of the metasoma, length occupying 0.69–0.83 of the metasoma. Coxae, femora, tibiae, and apices of tarsi brown, remainder pale yellow; tarsi 4-segmented.

**Male:** Antennal flagellum 11-segmented.

**Material examined:** 1♀1♂, County Road 312, Wujiaqu City, Xinjiang, N: 44°20’11.062”, E: 87°27’41.270”, H: 450.9 m, 08.vi.2023, reared from leaf galls on *Lycium ruthenicum*; 1♂, County Road 312, Wujiaqu City, Xinjiang, N: 44°20’11.062”, E: 87°27’41.270”, H: 450.9 m, 08.vi.2023, reared from leaf galls on *Lycium ruthenicum*; 1♀, Shigao Section, Manas County, Changji, Xinjiang, N: 44°30’35.075”, E: 86°23’52.919”, H: 398.5 m, 13.vi.2023, reared from leaf galls on *Lycium ruthenicum*; 1♂, Company 9, Regiment 103, Wujiaqu City, Xinjiang, N: 44°29’48.026”, E: 87°30’53.920”, H: 363.8 m, 20.vii.2023, reared from leaf galls on *Lycium ruthenicum*.

**Host:** *Aceria pallida* (Acari: Eriophyidae).

**Distribution:** China (Xinjiang).

Figure S1.7 Morphological characteristics of *Polynema haloxyloniae*

A: body, lateral view; B: thorax; C: hind wing; D: fore wing; E: antennae

**(5) *Trichogramma pintoi* Voegele, 1982**

Male: Body length 0.51–0.63 mm, pale brown. Compound eyes and ocelli red. Antennae pale yellow, 5-segmented; length ratio of scape : pedicel : funicle : clava 36:21:17:44. Midlobe of mesoscutum with a pale band. Ratio of forewing length to width 2.12–2.19:1; with pale brown infuscation below venation; venation "S"-shaped; ratio of submarginal vein : premarginal vein : stigmal vein 15:8:6; postmarginal vein absent; wing disc setose, marginal setae relatively short, ratio of longest marginal setae length to wing width 0.17–0.25:1. Hindwing narrow and long, with 3 tracks of setae; ratio of longest marginal setae length to wing width 1.56–1.63:1. Propodeum orange. Ratio of mesosomal length to metasomal length 0.72–0.81:1. Male genitalia: parameres bottle-shaped, narrow basally and broad apically, relatively slender; dorsal lamina relatively acute; aedeagus slightly longer than aedeagal apodemes. Coxae, mid-portions of femora, and apical tarsomeres pale brown, remainder pale yellow; tarsi 3-segmented.

Female: Female antennae 7-segmented, antennal formula 11221; ovipositor length approximately 0.84–0.91 times hind tibial length.

Material examined: 1♀, County Road 312, Wujiaqu City, Xinjiang, N: 44°20’11.062”, E: 87°27’41.270”, H: 450.9 m, 29.v.2023, reared from leaf galls on *Lycium ruthenicum*; 1♀1♂, County Road 312, Wujiaqu City, Xinjiang, N: 44°20’11.062”, E: 87°27’41.270”, H: 450.9 m, 04.vi.2023, reared from leaf galls on *Lycium ruthenicum*; 1♀, Shigao Section, Manas County, Changji, Xinjiang, N: 44°30’35.075”, E: 86°23’52.919”, H: 398.5 m, 09.vi.2023, reared from leaf galls on *Lycium ruthenicum*.

Host: Lepidopteran eggs; *Aceria pallida* (Acari: Eriophyidae).

Distribution: China (Inner Mongolia, Heilongjiang, Shanxi, Hainan, Xinjiang); Bulgaria, France, Portugal, Spain, Ukraine, Russia, Uzbekistan, Iran, India, Canada, USA, Cuba, Mexico, Argentina, Peru.

Figure S1.8 Morphological characteristics of *Trichogramma pintoi*

A: body, lateral view; B: abdomen; C: thorax; D: genitalia (♂); E: hind foot

**(6) *Aphelinoidea anatolica* Novicky, 1936**

**Female:** Body length 0.59–0.67 mm, pale yellowish-brown. Compound eyes and ocelli red. Head width slightly greater than head height. Antennal clava pale yellow, remainder pale brown; inserted slightly below the middle of the compound eyes; ratio of length to width for scape 3.47:1, pedicel 3.41:1, clava 3.92:1. Mandibles 3-dentate. Forewing broadly rounded, width 0.34–0.41 times length; venation pale yellow, relatively short, vein length 0.38–0.43 times wing length; postmarginal vein absent; wing disc densely covered with scattered setae, with a bare area; marginal setae relatively short, ratio of longest marginal setae length to wing width 0.19–0.24:1; with a dark brown spot below the stigmal vein. Hindwing narrow and long, with 3 tracks of setae; ratio of longest marginal setae length to wing width 2.01–2.79:1. Ratio of mesosomal length to metasomal length 0.72–0.81:1; ovipositor length 0.5 times metasomal length, extending from the fourth metasomal segment to the apex. Tarsi of all legs pale yellow, 3-segmented.

**Male:** Body color and main morphological characteristics similar to female.

**Material examined:** 1♀1♂, County Road 312, Wujiaqu City, Xinjiang, N: 44°20’11.062”, E: 87°27’41.270”, H: 450.9 m, 03.vi.2023, reared from leaf galls on *Lycium ruthenicum*; 1♂, County Road 312, Wujiaqu City, Xinjiang, N: 44°20’11.062”, E: 87°27’41.270”, H: 450.9 m, 06.vi.2023, reared from leaf galls on *Lycium ruthenicum*; 1♀, Shigao Section, Manas County, Changji, Xinjiang, N: 44°30’35.075”, E: 86°23’52.919”, H: 398.5 m, 05.vi.2023, reared from leaf galls on *Lycium ruthenicum*.

**Host:** *Aceria pallida* (Acari: Eriophyidae).

**Distribution:** China (Xinjiang); Turkey, Syria.

Figure S1.9 Morphological characteristics of *Aphelinoidea anatolica* Novicky

A: head; B: fore wing; C: hind wing; D: thorax; E: abdomen

**Parasitoid Complex of *Contarinia* sp.**

**(1) *Platygaster* sp.**

**Female:** Body length 1.24–1.38 mm, black. Antennae brown, setose; flagellum 8-segmented; length ratio of (radicle + scape) : pedicel : flagellum 153:48:298. Head in anterior view width 1.11–1.24 times length, 1.09–1.21 times mesosomal width. Occipital carina present. Ratio of mesosomal length to width 1.27–1.44:1; pronotum relatively narrow; mesoscutum with longitudinal rows of setae; posterolateral margins of mesoscutum, posterior margin of scutellum, metanotum, and propodeum densely covered with pubescence. Ratio of metasomal length to mesosomal length 1.14–1.42:1. Wings hyaline, venation reduced; forewing length 2.17–2.45 times width; hindwing with two hamuli, length 5.26–5.89 times width. Metasoma oval, length 1.74–2.12 times width. Legs brown, apices of all segments darkened.

**Male:** Morphology similar to female.

**Material examined:** 1♀, County Road 312, Wujiaqu City, Xinjiang, N: 44°20’11.062”, E: 87°27’41.270”, H: 450.9 m, 18.vii.2023, reared from flower bud galls on *Lycium ruthenicum*; 1♂, Company 9, Regiment 103, Wujiaqu City, Xinjiang, N: 44°29’48.026”, E: 87°30’53.920”, H: 363.8 m, 20.vii.2023, reared from leaf galls on *Lycium ruthenicum*; 1♂, County Road 312, Wujiaqu City, Xinjiang, N: 44°20’11.062”, E: 87°27’41.270”, H: 450.9 m, 24.viii.2023, reared from flower bud galls on *Lycium ruthenicum*; 1♀, Shigao Section, Manas County, Changji, Xinjiang, N: 44°30’35.075”, E: 86°23’52.919”, H: 398.5 m, 03.viii.2023, reared from flower bud galls on *Lycium ruthenicum*.

**Host:** *Contarinia* sp. (Diptera: Cecidomyiidae).

**Distribution:** China (Xinjiang).

Figure S1.10 Morphological characteristics of *Platygaster sp.*

A: body, dorsal view; B: antennae; C: thorax; D: abdomen
